# Neuroendocrine and Immune Responses Undertake Different Fates following Tryptophan or Methionine Dietary Treatment: Tales from a Teleost Model

**DOI:** 10.3389/fimmu.2017.01226

**Published:** 2017-09-27

**Authors:** Rita Azeredo, Marina Machado, António Afonso, Camino Fierro-Castro, Felipe E. Reyes-López, Lluis Tort, Manuel Gesto, Marta Conde-Sieira, Jesús M. Míguez, José L. Soengas, Eva Kreuz, Sven Wuertz, Helena Peres, Aires Oliva-Teles, Benjamin Costas

**Affiliations:** ^1^Centro Interdisciplinar de Investigação Marinha e Ambiental (CIIMAR), Novo Edifício do Terminal de Cruzeiros do Porto de Leixões, Matosinhos, Portugal; ^2^Departamento de Biologia, Faculdade de Ciências da Universidade do Porto (FCUP), Porto, Portugal; ^3^Instituto de Ciências Biomédicas Abel Salazar (ICBAS-UP), Universidade do Porto, Porto, Portugal; ^4^Department of Cell Biology, Physiology and Immunology, Universitat Autònoma de Barcelona, Barcelona, Spain; ^5^Laboratorio de Fisioloxía Animal, Departamento de Bioloxía Funcional e Ciencias da Saúde, Facultade de Bioloxía, Universidade de Vigo, Vigo, Spain; ^6^Department of Ecophysiology and Aquaculture, Leibniz-Institute of Freshwater Ecology and Inland Fisheries, Berlin, Germany

**Keywords:** methionine, tryptophan, innate immunity, inflammation, aquaculture, functional diets

## Abstract

Methionine and tryptophan appear to be fundamental in specific cellular pathways involved in the immune response mechanisms, including stimulation of T-regulatory cells by tryptophan metabolites or pro-inflammatory effects upon methionine supplementation. Thus, the aim of this study was to evaluate the immunomodulatory effect of these amino acids on the inflammatory and neuroendocrine responses in juveniles of European seabass, *Dicentrarchus labrax*. To achieve this, goal fish were fed for 14 days methionine and tryptophan-supplemented diets (MET and TRP, respectively, 2× dietary requirement level) or a control diet meeting the amino acids requirement levels (CTRL). Fish were sampled for immune status assessment and the remaining fish were challenged with intraperitoneally injected inactivated *Photobacterium damselae* subsp. *piscicida* and sampled either 4 or 24 h post-injection. Respiratory burst activity, brain monoamines, plasma cortisol, and immune-related gene expression showed distinct and sometimes opposite patterns regarding the effects of dietary amino acids. While neuroendocrine intermediates were not affected by any dietary treatment at the end of the feeding trial, both supplemented diets led to increased levels of plasma cortisol after the inflammatory insult, while brain monoamine content was higher in TRP-fed fish. Peripheral blood respiratory burst was higher in TRP-fed fish injected with the bacteria inoculum but only compared to those fed MET. However, no changes were detected in total antioxidant capacity. Complement factor 3 was upregulated in MET-fed fish but methionine seemed to poorly affect other genes expression patterns. In contrast, fish fed MET showed increased immune cells numbers both before and after immune challenge, suggesting a strong enhancing effect of methionine on immune cells proliferation. Differently, tryptophan effects on inflammatory transcripts suggested an inhibitory mode of action. This, together with a high production of brain monoamine and cortisol levels, suggests that tryptophan might mediate regulatory mechanisms of neuroendocrine and immune systems cooperation. Overall, more studies are needed to ascertain the role of methionine and tryptophan in modulating (stimulate or regulate) fish immune and neuroendocrine responses.

## Introduction

The neuroendocrine–immune interaction is an evolutionary conserved phenomenon among vertebrates. Two physiological responses evolved to help animals survive pathogen invasion and overcome situations of distress: the immune response and the stress response. In mammals, it has been recognized that both responses share some pathways (1). The importance of neuroendocrine–immune interactions in the physiological regulation of immune and brain functions is evident because vertebrate lymphoid tissue is innervated by parasympathetic and sympathetic nerve fibres which are implicated both in stimulation and inhibition of immune functions (2). Different hormones and cytokines, as well as their interactions, are involved in the same neuroendocrine–immune mechanisms (3). These interactions are especially evident in lower vertebrates such as fish in which, for instance, cytokines and neuropeptides are performing roles in both neuroendocrine and immune systems (4). Scientific evidence also shows that leucocytes are sensitive to a wide repertoire of neuroendocrine mediators. For instance, glucocorticoids influence the balanced successive secretion of pro- and anti-inflammatory cytokines and, in particular, cortisol can have profound and differential effects on the fish immune function (4).

Immune responses and stressful conditions can also affect amino acid requirements and metabolism (5). In fact, amino acid requirements may increase as direct consequence of metabolic changes associated with inflammation and infection (6). In fish, as in mammals, amino acids are versatile molecules displaying several functions besides protein constitution. For instance, amino acids have been shown to play important parts in immune mechanisms (7–10). Methionine has several features that make it a relevant amino acid during innate immune responsive mechanisms. Methionine appears to have clear pro-inflammatory effects on juvenile carp, *Cyprinus carpio* (11, 12). *S*-adenosyl-methionine directly participates in polyamine biosynthesis by successively adding aminopropane to the forming polyamines, important elements to cell proliferation (13). In addition, free radical scavengers, such as melatonin or glutathione, control the increased oxidative damage caused by the production of reactive oxygen or nitrogen species. Methionine is precursor of cysteine, which is one of the three glutathione constituents, and thus an essential element for its production.

Understanding the regulatory mechanisms of the immune system is necessary upon the analysis of a particular immune response. According to Frumento et al. (14), Grohmann et al. (15), and Le Floc’h et al. (16), the role of tryptophan during the immune response is mainly related to regulatory processes. As substrate of 2, 3-indoleamine dioxygenase (IDO), tryptophan metabolic cascades lead to anti-inflammatory signaling molecules, the main effectors being regulatory T-cells. Considering this, an increase in the tryptophan availability in the organism may be regarded as a strategy to counteract the deleterious aspects of an eventual exacerbated innate immune response. However, considering the existing link between the immune and the neuroendocrine systems mentioned above, tryptophan effects must also be evaluated from an alternative perspective. Serotonin (5-HT) is a monoamine neurotransmitter that is produced from tryptophan in the central nervous system and its synthesis is known to control the adrenocorticotropic hormone release, which in turn directly regulates cortisol production (17). The few available studies in fish have shown that the teleost immune response, like that of mammals, is sensitive to 5-HT-induced immunomodulation (18). Lepage et al. (19) observed that tryptophan supplementation in juvenile rainbow trout, *Oncorhynchus mykiss*, had opposed effects on cortisol levels, which were slightly increased in non-stressed fish, while were reduced in stressed fish.

Increasing evidence shows that dietary supplementation of specific amino acids to animals and humans with infectious diseases enhances the immune status, thereby reducing morbidity and mortality (9). In fish, the immunomodulatory role of amino acids has only recently started to be studied, and the underlying cellular and molecular mechanisms still need to be unfold. Therefore, the main goal of this study was to gather evidence on the specific fate of amino acids arbitrating the elaborate neuroendocrine–immune network. The present study focused on several neuroendocrine and immunological aspects of sham or antigen-stimulated animals, using the European seabass (*Dicentrarchus labrax*) as model species. Particular emphasis was given to the study of monoamine responses in specific brain regions, the expression patterns of a panel of immune-related genes in both the head-kidney (HK) and blood, as well as peripheral blood cell dynamics in which the respiratory burst was determined.

## Materials and Methods

### Formulation and Analytical Procedures with Experimental Diets

Three isonitrogenous (44.9% crude protein) and isolipidic (14.9% crude fat) diets were formulated. Fish meal and a blend of plant feedstuffs were used as protein sources, whereas fish oil was the main fat source. The plant-protein fraction represented almost 50% of the total feed composition. In two of these diets, l-tryptophan or l-methionine was added at the expenses of fish meal to a final concentration of 2× the requirement level determined for seabass. A non-supplemented diet was used as a control diet (CTRL) meeting the amino acid requirement of European seabass (20). The two supplemented diets were considered dietary treatments and will be referred to as TRP (tryptophan-supplemented diet) and MET (methionine-supplemented diet).

More detailed information on diets composition and proximate analysis is given in Table 1. All ingredients were ground, mixed together, and dry-pelleted in a laboratory pellet mill (CPM, California Pellet Mill, Crawfordsville, IN, USA). Proximate analysis of the diets was performed according to the Association of Official Analytical Chemists methods (21) and amino acids analysis was carried out according to Banuelos-Vargas et al. (22) (Table 2).

**Table 1:** Ingredients and proximate composition of experimental diets as percentage of dry matter (% DM).

	Experimental diets		
	CTRL	TRP	MET
**Ingredients (% DM)**			

Fish meal^a^	34.1	33.5	33.2
Soybean meal^b^	15.0	15.0	15.0
Corn gluten^c^	10.0	10.0	10.0
Wheat gluten^d^	5.0	5.0	5.0
Wheat meal^e^	16.7	16.6	16.2
Fish oil	13.9	13.4	14.0
Vitamin premix^f^	1.0	1.0	1.0
Choline chloride (50%)	0.5	0.5	0.5
Mineral premix^g^	1.0	1.0	1.0
Binder^h^	1.0	1.0	1.0
Agar	1.0	1.0	1.0
Dibasic calcium phosphate	0.84	0.91	0.96
l-Methionine^i^	–	–	1.16
l-Tryptophan^i^	–	0.52	–

**Proximate analyses (% dry weight)**			

DM (%)	95.2	94.9	94.3
Crude protein	44.9	45.2	45.0
Crude lipid	15.5	16.5	16.9
Ash	10.5	10.4	10.5

*^a^Pesquera Centinela, Steam Dried LT, Chile (CP: 71.4%; CL 9.3%). Sorgal, S.A. Ovar, Portugal*.

*^b^Soybean meal (CP: 54.9%; CL:2.1%), Sorgal, S.A. Ovar, Portugal*.

*^c^Corn gluten (CP: 72.2%; CL: 2.0%), Sorgal, S.A. Ovar, Portugal*.

*^d^Wheat gluten (CP: 84.4%; CL: 2.1%), Sorgal, S.A. Ovar, Portugal*.

*^e^Wheat meal (CP: 13.9%; CL: 1.8%), Sorgal, S.A. Ovar, Portugal*.

*^f^Vitamins (mg kg^−1^ diet): retinol, 18,000 (IU kg^−1^ diet); calciferol, 2,000 (IU kg^−1^ diet); alpha tocopherol, 35; menadion sodium bis., 10; thiamin, 15; riboflavin, 25; Ca pantothenate, 50; nicotinic acid, 200; pyridoxine, 5; folic acid, 10; cyanocobalamin, 0.02; biotin, 1.5; ascorbyl monophosphate, 50; inositol, 400*.

*^g^Minerals (mg kg^−1^ diet): cobalt sulfate, 1.91; copper sulfate, 19.6; iron sulfate, 200; sodium fluoride, 2.21; potassium iodide, 0.78; magnesium oxide, 830; manganese oxide, 26; sodium selenite, 0.66; zinc oxide, 37.5; dicalcium phosphate, 8.02 (g kg^−1^ diet); potassium chloride, 1.15 (g kg^−1^ diet); sodium chloride, 0.4 (g kg^−1^ diet)*.

*^h^Aquacube. Agil, UK*.

*^i^Feed grade amino acids, Sorgal, SA. Ovar, Portugal*.

**Table 2:** Amino acid composition (g 16 g^−1^ N) of the experimental diets determined as described.

	Experimental diet		
	CTRL	TRP	MET
Arginine	7.74	7.11	7.02
Histidine	3.78	3.63	4.12
Isoleucine	5.05	4.64	4.71
Leucine	9.73	9.71	9.49
Lysine	6.66	6.96	6.68
Methionine	2.57	2.42	4.95
Phenylalanine	5.39	5.16	5.16
Tyrosine	4.04	3.96	3.98
Threonine	4.68	4.38	4.51
Tryptophan	1.12	2.24	1.10
Valine	5.38	5.10	5.12
Aspartic acid	8.20	7.54	7.60
Glutamic acid	16.39	16.14	16.09
Serine	4.15	4.42	4.29
Glycine	3.98	4.18	4.20
Alanine	4.89	4.92	4.97
Proline	4.99	5.50	4.84

### Bacteria Inoculum Preparation

*Photobacterium damselae* subsp. *piscicida*, strain PP3 (*Phdp*), isolated from Japanese amberjack (*Seriola quinqueradiata*, Japan) by Dr. Andrew C. Barnes (Marine Laboratory, Aberdeen, UK) and kindly provided by Dr. Ana do Vale (Institute for Molecular and Cell Biology, University of Porto, Portugal). Bacteria were first cultured for 48 h at 22°C in tryptic soy agar (Difco Laboratories) supplemented with 1% NaCl (w/v) (TSA-1). Colonies were then inoculated into tryptic soy broth equally supplemented with NaCl (TSB-1) and incubated overnight at 22°C. Bacteria under exponentially growth were centrifuged at 3,500 × *g* for 30 min, resuspended in TSB-1 with glycerol at a final concentration of 15% (v/v), and stored at −80°C as a stock solution. *Phdp* inoculum was obtained by culturing bacteria from the stock solution as previously described and by suspending in sterile Hank’s Balanced Salt Solution (HBSS) at a final concentration of 1 × 10^6^ colony forming units ml^−1^, according to Costas et al. (23). Bacteria were killed by 2 h UV-light exposure. No bacterial growth was observed when UV-killed bacteria were plated on TSA-1.

### Fish and Experimental Design

This study was carried out at the Marine Zoological Station, Porto, Portugal. After 2 weeks of acclimatization, juvenile European seabass (274.7 ± 20.4 g) fed a commercial diet were randomly distributed in six fiberglass tanks (300 l; *n* = 15) in a seawater recirculation system (temperature: 25 ± 1°C; salinity: 35 ppt; natural light–dark cycle). Dietary treatments were randomly assigned to duplicate tanks and fish were fed twice a day until apparent satiety. The feeding trial lasted for 14 days, and O_2_, salinity, pH, temperature, and nitrogenous compounds were monitored daily. At the end of the feeding trial, three fish per tank (six fish per dietary treatment, *n* = 6) were euthanized by immersion in 2-phenoxyethanol (1,500 ppm; Sigma) and sampled. Blood was collected from the caudal vessel with heparinized syringes and kept in heparinized tubes at 4°C until analyzed. The HK was removed and kept in RNAlater^®^ solution (Ambion Inc., Austin, TX, USA) at 4°C for 24 h and then stored at −80°C until assayed. Liver and brain were collected was well and brain was dissected in the following regions: hypothalamus, optic tectum, and telencephalon (including olfactory bulb). Both liver and brain samples were immediately frozen in dry ice and later stored at −80°C.

Thereafter, the remaining fish (12 fish per tank) were intraperitoneally (i.p.) injected with either 100 µl of UV-killed *Phdp* or HBSS (sham group), according to an inflammatory model previously established (24) and redistributed into new tanks according to dietary treatment and stimuli. Afterward, fish were euthanized and sampled (six fish per dietary treatment, per sampling time, *n* = 6) either 4 or 24 h post-inoculation as mentioned above.

The experiments were approved by the Animal Welfare Committee of the Interdisciplinary Centre of Marine and Environmental Researcha and carried out in a registered installation (N16091.UDER). Experiments were performed by trained scientists in full compliance with national rules and following the European Directive 2010/63/EU of the European Parliament and the European Union Council on the protection of animals used for scientific purposes.

### Analytical Procedures with Blood and Peritoneal Leukocytes

An aliquot of gently homogenized blood was used to perform total white blood cells (WBC) counts. The remaining blood was centrifuged at 10,000 × *g* for 10 min at 4°C and plasma stored at −80°C until assayed. Blood smears were air dried and stained with Wright’s stain (Haemacolor; Merck) after fixation with formol-ethanol (10% of 37% formaldehyde in absolute ethanol). The slides were examined (1,000×). At least 200 leukocytes were counted per smear and classified as thrombocytes, lymphocytes, monocytes, and neutrophils. Detection of peroxidase activity was carried out as described by Afonso et al. (24) in order to facilitate identification of neutrophils. The relative percentage and absolute value (×10^4^ ml^−1^) of each cell type was subsequently calculated.

#### Respiratory Burst in Blood Leukocytes

The respiratory burst in peripheral leukocytes was evaluated according to Nikoskelainen et al. (25) with some modifications. Briefly, 4 µl of blood were added to 96 µl of HBSS in a 96-well flat bottom, white polystyrene plate. Afterward, 100 µl of freshly prepared luminol solution (2 mM luminol in 0.2 M borate buffer pH 9.0, with 2 µg ml^−1^ phorbol 12-myristate 13-acetate) were added to each well. Luminol-amplified chemiluminescence was measured every 3 min for 2 h in a luminescence reader (BioTek Synergy HT microplate reader) for generation of kinetic curves. Each sample was run in triplicate and controls contained no blood. The integral luminescence in relative light units was calculated.

#### Peritoneal Leukocytes

The peritoneal cells were collected according to a procedure initially described for mice (26) and posteriorly adapted for fish (27). Briefly, following anesthesia and blood collection, 5 ml of cold HBSS supplemented with 30 U ml^−1^ heparin were injected into the peritoneal cavity. Afterward, the peritoneal area was slightly massaged in order to disperse the peritoneal cells in the injected HBSS. The HBSS containing the suspended cells was then collected. Total peritoneal cell counts were performed with a hemocytometer. Cytospin preparations were then made with a THARMAC Cellspin apparatus and stained as described above for blood smears. The lymphocytes, macrophages, and neutrophils in the peritoneal exudates were counted and the percentage of each cell type was established after counting a minimum of 200 cells per slide. The concentration (×10^4^ ml^−1^) of each leukocyte type was also calculated.

### Liver Total Antioxidant Capacity (TAC)

Liver samples were freeze-dried for 48 h (Alpha 1–4 LOC-1M, Christ) and homogenized with a TissueLyser (Qiagen). Tris–HCl buffer [50 mM Tris–HCl pH 7.5, 4 mM EDTA, 50 mM NaF, 0.5 mM phenylmethylsulfonyl fluoride, 1 mM dithiothreitol, 250 mM sucrose] was added to powdered frozen liver at a ratio of 5:1 v/w. After vortexing, samples were centrifuged at 20,000 × *g* for 30 min, at 4°C, and the supernatant was transferred to a new vial.

Total protein content was determined in duplicate according to Bradford (28) using the Roti^®^-Quant reagent and a bovine serum albumin BSA dilution series (29). Briefly, 50 µl of supernatant were diluted with water (1:300). Then, 200 µl of Roti^®^-Quant reagent (1×) were added to each well and the plate was incubated for 10 min at room temperature, protected from light. Absorbance was read at 595 nm with a microplate Infinite 200 reader (TECAN).

Total antioxidant capacity was analyzed to quantify the activity of both antioxidant proteins and smaller molecules using a TAC assay kit (Sigma) as described before. In brief, supernatant was diluted with phosphate buffer (1:250) and 100 µl of the diluted sample with 100 µl of a Cu^2+^ working solution, mixed and incubated for 90 min at room temperature. The absorbance was read at 570 nm. TAC was determined in duplicate with a Trolox standard curve and concentration calculated based on the protein content.

### Brain Monoamine Content

The brain content of noradrenaline (NA), dopamine (DA), 3,4-dihydroxyphenylacetic acid (DOPAC, a major DA metabolite), 5-HT, and 5-hydroxyindoleacetic acid (5-HIAA) were analyzed by high performance liquid chromatography with electrochemical detection as previously described by Gesto et al. (30). Briefly, tissues were homogenized by ultrasonic disruption in 0.5 ml of mobile phase with the following composition: 85 mM sodium hydrogen phosphate, 0.72 mM octanosulfonic acid, 18% methanol, and adjusted to pH 3.0. Homogenates were centrifuged (16,000 × *g* for 10 min at room temperature) and prior to analysis supernatants were diluted 1:1 (supernatant/mobile phase) for optic tectum and 1:2 for telencephalon and hypothalamus. A 20-µl aliquot of each sample was injected into the HPLC system consisting of a Jasco PU2080 pump equipped with a Jasco AS-2057 autosampler, and an ESA Coulochem II detector (Bedford, MA, USA). The detection system included a M5011 ESA analytical cell with electrode potentials set at +20 mV and +300 mV, respectively. All separations were performed at room temperature at a flow rate of 8 ml/min. Acquisition and integration of chromatograms were performed by using the ChromNAV version 1.12 software (Jasco Corp.).

### Plasma Cortisol

Cortisol was extracted from 20 µl of plasma in 180 µl of diethyl ether (Sigma). Cortisol levels in plasma were determined using a commercial ELISA kit (RE52061, IBL International GMBH, Germany), with a sensitivity of 0.05 ng/ml and intra- and inter-assay coefficients of variation of 2.98 and 3.51%, respectively. This kit was previously validated for teleosts (31) and to validate this test for European seabass plasma samples two different tests were performed: dilution parallelism and recovery. The dilution parallelism test consisted of four consecutive dilutions of a European seabass plasma sample with high and known concentration of cortisol, which was then compared with the standard curve. The curve obtained from seabass plasma was parallel to the standard curve, thus validating the test for this species (results not shown). The recovery test consisted of adding increasing known amounts of cortisol to a European seabass plasma sample, using kit standards (standards D, E, and F). A recovery value of 84.5 ± 11.9% was obtained, reinforcing the validity of the kit for European seabass. Main cross-reactivities (>1%; given by the supplier) were 30% for prednisolone, 11% for 11-Desoxy-Cortisol, 4.2% for cortisone, 2.5% for prednisone, and 1.4% for corticosterone. Since cortisol is the main steroid produced by fish interrenal tissue, cross-reactivity with other steroids was assumed negligible.

### Immune-Related Gene Expression in HK

For HK immune-related gene expression, RNA was extracted with Tri Reagent (Sigma) following the manufacturer’s instructions, and resuspended in free nuclease water (Invitrogen). RNA was quantified using a NanoDrop-1000 spectrophotometer (Thermo Scientific). Total RNA per sample (3 µg) was used for cDNA synthesis, which was performed using a SuperScript^®^ III Reverse Transcriptase kit (Invitrogen) and Oligo-dT primer (Promega), according to manufacturer’s instructions.

A set of different primers was designed to evaluate immune-relevant gene expression profiles. The chosen genes were: interleukin-1β (*il1*β), matrix-metalloproteinase 9 (*mmp9*), glutathione peroxidase (*gpx*), complement factor 3 (*c3*), hepcidin (*hamp*), glucocorticoid receptor (*gr*), and melanocortin 2 receptor (*mc2r*). Primer sequences were designed with Primer-Blast software (NCBI) and the structural primer analysis was performed with Oligoanalyzer (IDT^®^). Primer sequences are listed in Table 3.

**Table 3:** Specifications of real-time PCR assays including forward (F) and reverse (R) primers, length of amplicon, GenBank ID (NCBI), annealing temperature, and PCR efficiency.

Gene	Acronym	GenBank ID	Eff (%)^a^	Ta (°C)	Amplicon length (bp)	Primer sequence (5′–3′)
Elongation factor 1α	*ef1*α	AJ866727.1	96.5	57	144	F: AACTTCAACGCCCAGGTCAT
						R: CTTCTTGCCAGAACGACGGT

40S ribosomal protein SA	*rpsa*	HE978789.1	93.0	55	79	F: TGATTGTGACAGACCCTCGTG
						R: CACAGAGCAATGGTGGGGAT

Interleukin-1β	*il1*β	AJ269472.1	96.7	57	105	F: AGCGACATGGTGCGATTTCT
						R: CTCCTCTGCTGTGCTGATGT

Matrix metalloproteinase 9	*mmp9*	FN908863.1	98.4	57	166	F: TGTGCCACCACAGACAACTT
						R: TTCCATCTCCACGTCCCTCA

Complement factor 3	*c3*	HM563078.1	111.5	57	165	F: CAGTGGGAATCTGTGGGCTT
						R: GGCAAACACCTTGGCAAC

Glutathione peroxidase	*gpx*	DT044993	94.2	57	176	F: GTTTGGACATCAGGAGAACTGC
						R: CATCGCTGGGGTATGGAAGC

Hepcidin	*hamp*	DQ131605.1	94.2	57	172	F: CTGGAGGAGCCAATGAGCAA
						R: TGGAGAGAGCATCAGAGCAC

Glucocorticoid receptor	*gr*	AY549305.1	111.2	55	110	F: AGGCATTCACCACCCCATTC
						R: GAAGTGACCCAGGCTGTTGA

Melanocortin 2 receptor	*mc2r*	FR870225.1	108.7	55	676	F: GGAACAGGAACCTCCACTCG
						R: ACCACGTGTAGCTGGAACAG

Dicentracin	*dctrn*	AY303949.1	89.2	55	70	F: CTCATGGCTGAACCTGGGG
						R: TGGACTTGCCGACGTGAAC

*^a^Efficiency of PCR reactions (represented in percentage) were calculated from serial dilutions of tissue RT reactions in the validation procedure*.

To set up the real-time PCR, the optimal amplification condition of each primer pair was standardized by a temperature annealing gradient in conventional PCR. Efficiency of each primer pair was determined by real-time PCR according to Pfaffl (32). Quantitative PCR reactions were carried out in a CFX384 Touch™ Real-Time PCR Detection System. Each reaction contained 1 µl of diluted cDNA (1:5 dilution) mixed with 6.25 µl of iTaq™ Universal SYBR^®^ Green (BioRad) and 0.25 µl (10 mM) of each specific primer in a final volume of 12.5 µl. The thermal conditions used were 3 min at 95°C of pre-incubation, followed by 40 cycles at 95°C for 10 s and 57°C (annealing temperature) for 30 s. Melting curve analysis was always performed to confirm specificity of the reaction. The expression of the target genes was normalized using the elongation factor 1α (*ef1*α) gene of European seabass. Data was standardized by dividing the normalized expression values of cells from different treatments by the normalized expression values of the control group.

### Immune-Related Gene Expression in Whole Blood

For whole blood immune-related gene expression, RNA was extracted with Tri Reagent (Sigma) following the manufacturer’s instructions, and resuspended in free nuclease water (Invitrogen). RNA was quantified using Take 3 Microvolume Plate (Biotek) and samples were then treated with DNase using the RQ1 RNase-free DNase kit (Promega) following manufacturer’s indications. Total RNA per sample (1.5 µg) was used for cDNA synthesis, which was performed using the NZY First-Strand cDNA Synthesis Kit (NZYTech) according to the manufacturer’s instructions.

A set of different primers was designed to evaluate immune-relevant gene expression profiles. The chosen genes were: *il1*β, *mmp9, gpx*, dicentracin (*dctrn*), and *gr*. Primer sequences were designed with Primer-Blast software (NCBI) and the structural primer analysis was performed with Oligoanalyzer (IDT^®^). Primer sequences are listed in Table 3. Optimization of assays was carried out as described above.

Quantitative PCR reactions were carried out in an Eppendorf Mastercycle ep realplex. Each reaction contained 1 µl of diluted cDNA (1:5 dilution) mixed with 10 µl of NZYSpeedy qPCR Master Mix and 0.4 µl (10 mM) of each specific primer in a final volume of 20 µl. The thermal conditions used were 10 min at 95°C of pre-incubation, followed by 40 cycles at 95°C for 15 s and annealing temperature for 1 min. Melting curve analysis was always performed to confirm specificity of the reaction. The expression of the target genes was normalized using the 40S ribosomal protein SA (*rpsa*) gene of European seabass. Standardization was carried out as described above.

### Data and Statistical Analysis

Statistical analyses were performed with STATISTICA (StatSoft, Inc. 2013, version 12) for WINDOWS. Results are expressed as means ± SD of the mean. Data were analyzed for normality and homogeneity of variance and, when necessary, outliers were removed using the STATISTICA tool for outliers and extremes removal. Data were log-transformed and analyzed by one-way analysis of variance (ANOVA) (immune status-related analysis) or Multifactorial ANOVA (inflammatory response) with dietary treatment and sampling time as variables. Whenever significant differences were found among groups, a multiple-comparisons Tukey HSD test was performed to identify significantly different groups. For every test, the level of significance chosen was *p* ≤ 0.05.

## Results

### Neuroendocrine Response

#### Brain Monoamine Content

##### Hypothalamus

At the end of the feeding trial, NA content was higher in MET-fed fish than in those fed TRP (Table S1 in Supplementary Material). After being i.p.-injected, the 5-HIAA/5-HT ratio was higher in fish sampled at 4 h than in those sampled at 24 h post-injection, regardless dietary treatment (Table S1 in Supplementary Material).

##### Telencephalon

**Figure 1:**
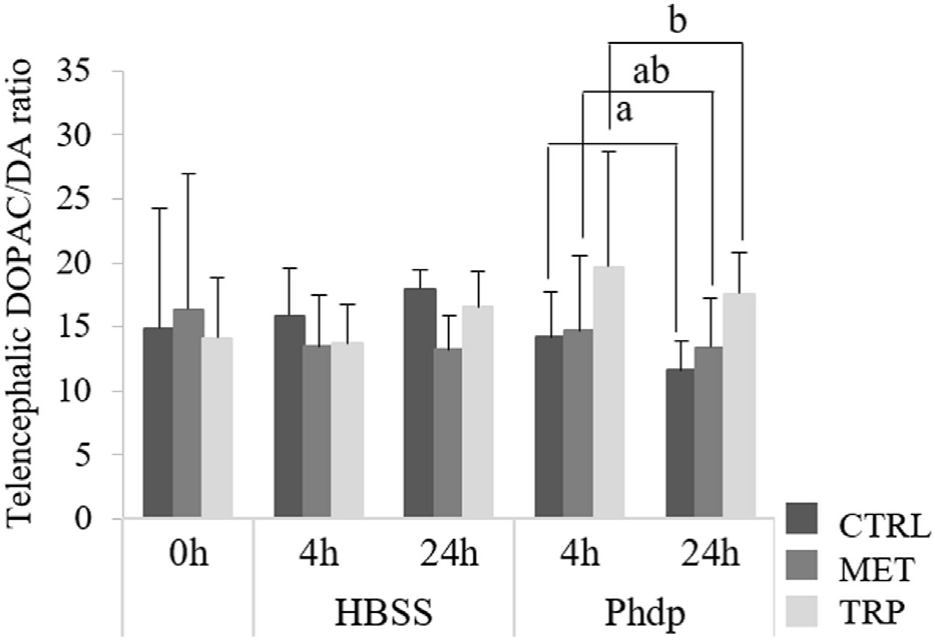
Telencephalic dihydroxyphenylacetic acid (DOPAC)/dopamine (DA) ratio in juvenile European seabass fed CTRL (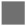), MET (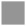) or TRP (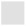) for 14 days (0 h) and sampled at 4 h or 24 h post Hank’s balanced salt solution (HBSS) or *Phdp* i.p. injection (mean ± SD, *n* = 6). a and b stand for significant differences between dietary treatments. One-way analysis of variance (ANOVA) (before i.p. injection) and multifactorial ANOVA (after i.p. injection); Tukey *post hoc* test; *p* ≤ 0.05.

No effects were observed in monoamine content before i.p. injection (Table S2 in Supplementary Material). However, DA content decreased at 24 h post-injection in HBSS-injected fish fed CTRL (Table S2 in Supplementary Material). Moreover, DOPAC levels decreased over time and were lower in *Phdp*-injected fish than in their HBSS counterparts, regardless of dietary treatment and sampling time (Table S2 in Supplementary Material). 5-HT and 5-HIAA levels were higher in TRP-fed fish than in MET-fed fish but no differences were observed compared to the CTRL group (Table S2 in Supplementary Material). Fish fed TRP showed higher DOPAC/DA ratio compared to the CTRL-fed group but only in *Phdp*-injected animals (Figure 1).

##### Optic Tectum

**Figure 2:**
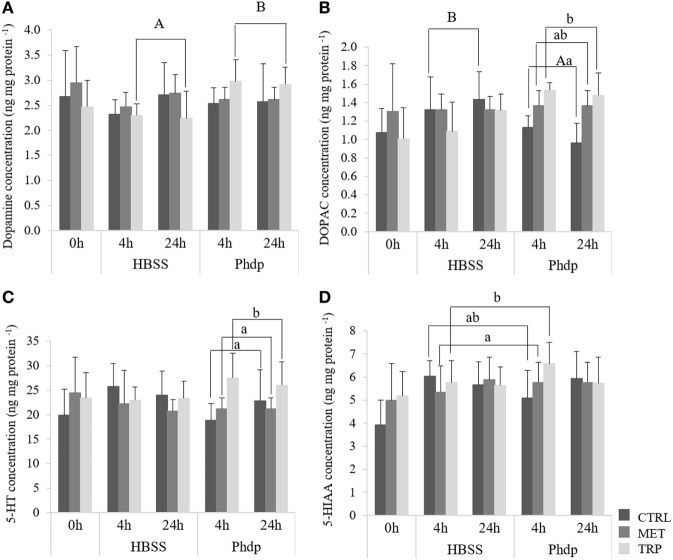
Optic tectum dopamine [DA, **(A)**], 3,4-dihydroxyphenylacetic acid [dihydroxyphenylacetic acid (DOPAC), **(B)**] serotonin [5-HT, **(C)**], and 5-hydroxyindoleacetic acid (5-HIAA). **(D)** content in juvenile European seabass fed CTRL (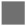), MET (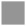), or TRP (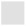) for 14 days (0 h) and sampled at 4 h or 24 h post HBSS or *Phdp* i.p. injection (mean ± SD, *n* = 6). a and b stand for significant differences attributed to dietary treatments. A and B depict differences attributed to stimuli. One-way analysis of variance (ANOVA) (before i.p. injection) and Multifactorial ANOVA (after i.p. injection); Tukey *post hoc* test; *p* ≤ 0.05.

No effects were observed in monoamine content before i.p. injection (Table S3 in Supplementary Material). Following bacterial inoculation, DA was observed to be higher in *Phdp*-injected fish fed TRP than in the correspondent HBSS-injected group, regardless of sampling time (Figure 2A). DOPAC was higher in CTRL-fed fish injected with HBSS than in bacteria-injected fish, regardless of sampling time (Figure 2B). Moreover, TRP-fed fish injected with *Phdp* showed higher DOPAC levels than their CTRL counterparts (Figure 2B). 5-HT was higher in TRP-fed fish compared to both CTRL- and MET-fed fish, but only in *Phdp*-injected group (Figure 2C) while 5-HIAA was also higher in TRP-fed fish but only compared to those fed MET and sampled at 4 h post-injection (Figure 2D).

#### Plasma Cortisol

**Figure 3:**
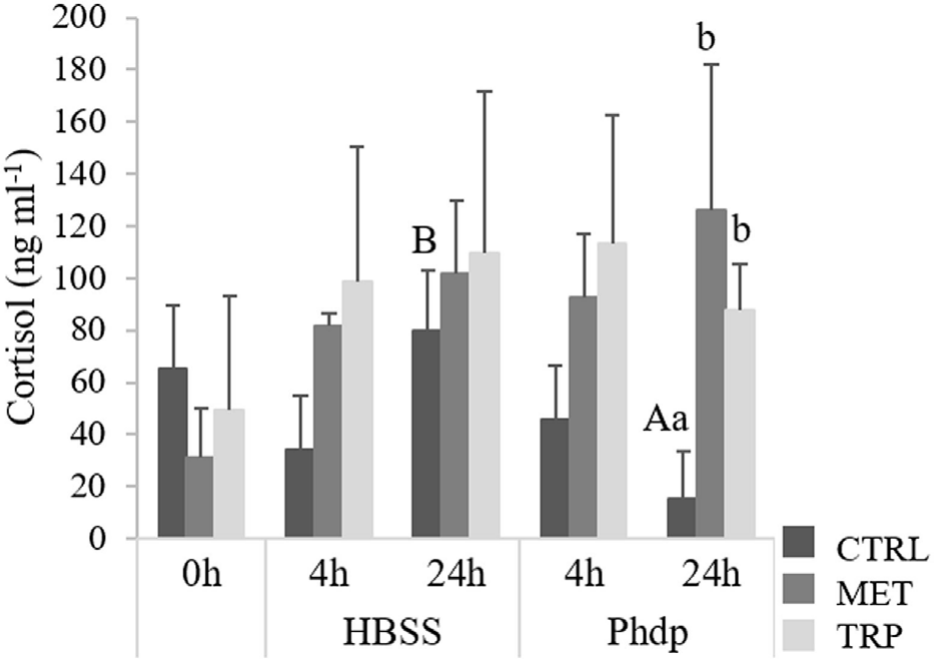
Plasma cortisol levels in juvenile European seabass fed CTRL (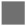), MET (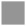), or TRP (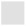) for 14 days (0 h) and sampled at 4 h or 24 h post Hank’s balanced salt solution (HBSS) or *Phdp* i.p. injection (mean ± SD, *n* = 6). a and b stand for significant differences attributed to dietary treatments. A and B depict differences attributed to stimuli. One-way analysis of variance (ANOVA) (before i.p. injection) and multifactorial ANOVA (after i.p. injection); Tukey *post hoc* test; *p* ≤ 0.05.

Fish fed the experimental diets for 14 days showed no differences on plasma cortisol levels (Figure 3). Cortisol levels were higher in *Phdp*-injected fish fed supplemented diets and sampled at 24 h post-injection than in CTRL-fed counterparts (Figure 3). Moreover, in CTRL-fed fish, cortisol was higher in HBSS-injected fish sampled at 24 h post-injection than in those injected with *Phdp*.

#### Quantitative *gr* and *mc2r* mRNA Expression

**Figure 4:**
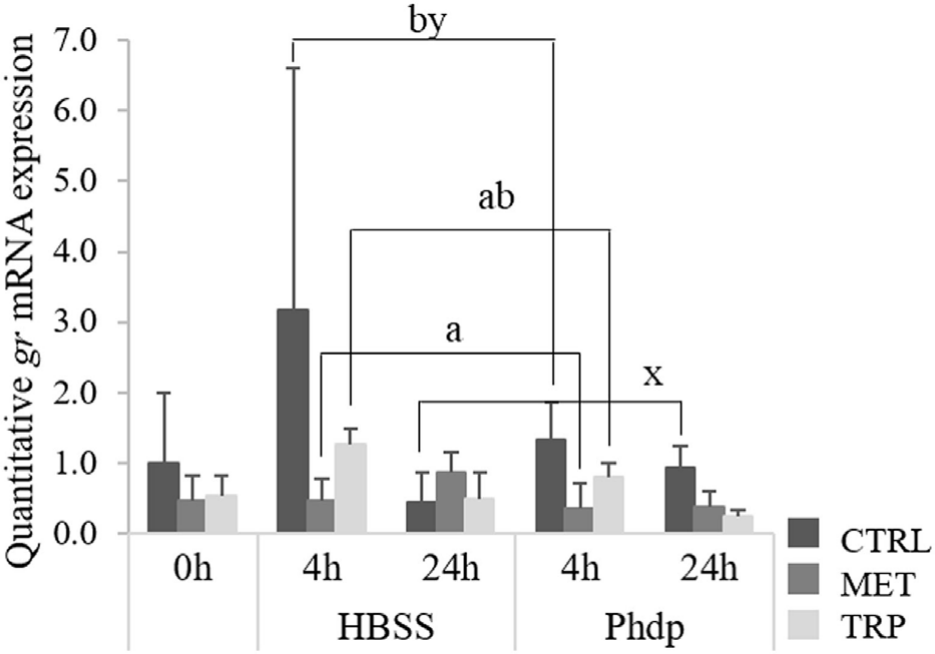
Quantitative *gr* mRNA expression in blood of juvenile European seabass fed CTRL (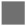), MET (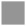), or TRP (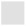) for 14 days (0 h) and sampled at 4 or 24 h post Hank’s Balanced Salt Solution (HBSS) or *Phdp* i.p. injection (mean ± SD, *n* = 6). x and y stand for significant differences attributed to sampling time. a and b stand for significant differences attributed to dietary treatments. One-way analysis of variance (ANOVA) (before i.p. injection) and multifactorial ANOVA (after i.p. injection); Tukey *post hoc* test; *p* ≤ 0.05.

Expression levels of *gr* and *mc2r* in the HK were not affected by any dietary treatments (Table S5 in Supplementary Material). However, mRNA levels of both receptors were downregulated over time, after i.p. injection (Table S5 in Supplementary Material). Differently, *gr* was downregulated in blood of CTRL-fed fish sampled at 24 h compared to the same dietary group sampled at 4 h post-injection, regardless of stimulus (Figure 4). Moreover, *gr* expression levels were lower in MET-fed fish compared to the CTRL-fed group sampled at 4 h post-injection (Figure 4).

### Oxidative Stress

#### Liver TAC

No significant differences were observed in TAC at the end of the feeding trial but it increased in time after i.p. injection regardless of dietary treatment and stimulus (Table S4 in Supplementary Material).

#### Respiratory Burst

**Figure 5:**
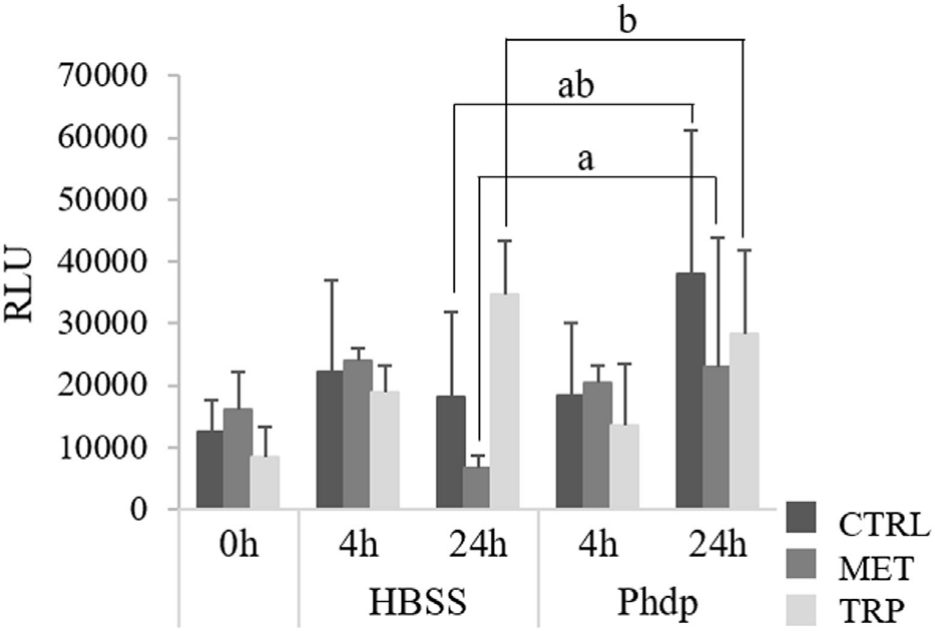
Respiratory burst activity of peripheral leukocytes in juvenile European seabass fed CTRL (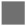), MET (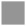), or TRP (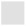) for 14 days (0 h) and sampled at 4 or 24 h post Hank’s balanced salt solution (HBSS) or *Phdp* i.p. injection (mean ± SD, *n* = 6). a and b stand for significant differences attributed to dietary treatments. One-way analysis of variance (ANOVA) (before i.p. injection) and multifactorial ANOVA (after i.p. injection); Tukey *post hoc* test; *p* ≤ 0.05.

Peripheral blood superoxide anion production was not affected by dietary treatments at the end of the feeding trial (Figure 5). However, at 24 h post-injection, superoxide production was highest in TRP-fed fish, though values were only significantly higher than those measured in MET-fed fish (Figure 5).

#### Relative HK and Blood *gpx* mRNA Expression

**Figure 6:**
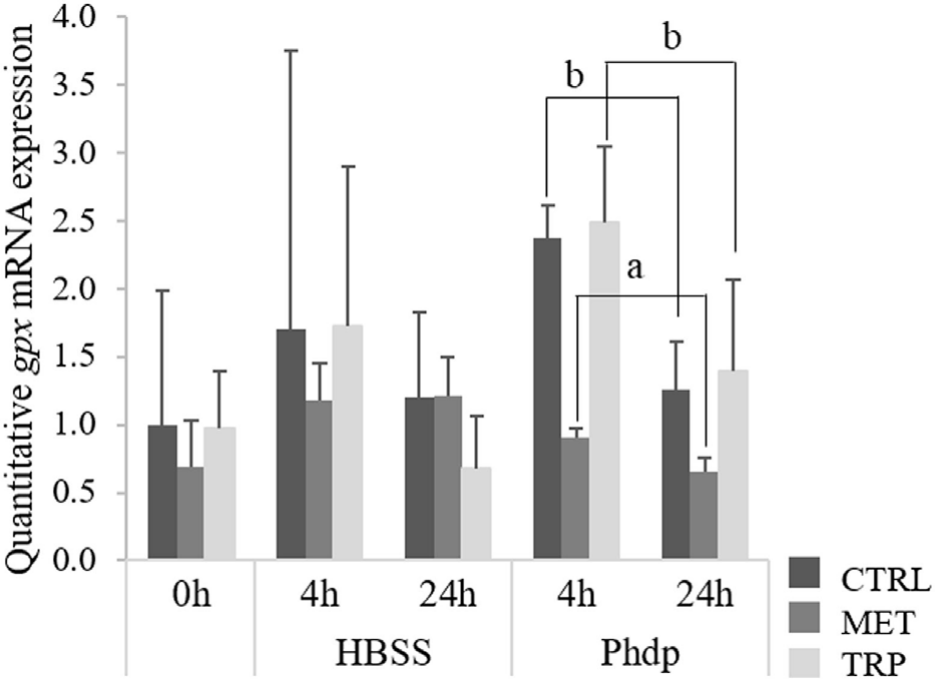
Quantitative *gpx* mRNA expression in blood of juvenile European seabass fed CTRL (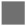), MET (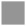), or TRP (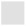) for 14 days (0 h) and sampled at 4 or 24 h post Hank’s balanced salt solution (HBSS) or *Phdp* i.p. injection (mean ± SD, *n* = 6). a and b stand for significant differences between dietary treatments. One-way analysis of variance (ANOVA) (before i.p. injection) and Multifactorial ANOVA (after i.p. injection); Tukey *post hoc* test; *p* ≤ 0.05.

At the end of the feeding trial, *gpx* was up-regulated in the HK of fish fed both supplemented diets relatively to the CTRL-fed group, while those fed TRP showed higher expression levels than MET-fed fish, too (Table S5 in Supplementary Material). Differently, expression levels of *gpx* in blood were lower in MET-fed fish sampled at 24 h post-injection relatively to those of CTRL- and TRP-fed fish (Figure 6).

### Immune Response

#### Leukocyte Migration Dynamics

##### Peripheral Blood

**Figure 7:**
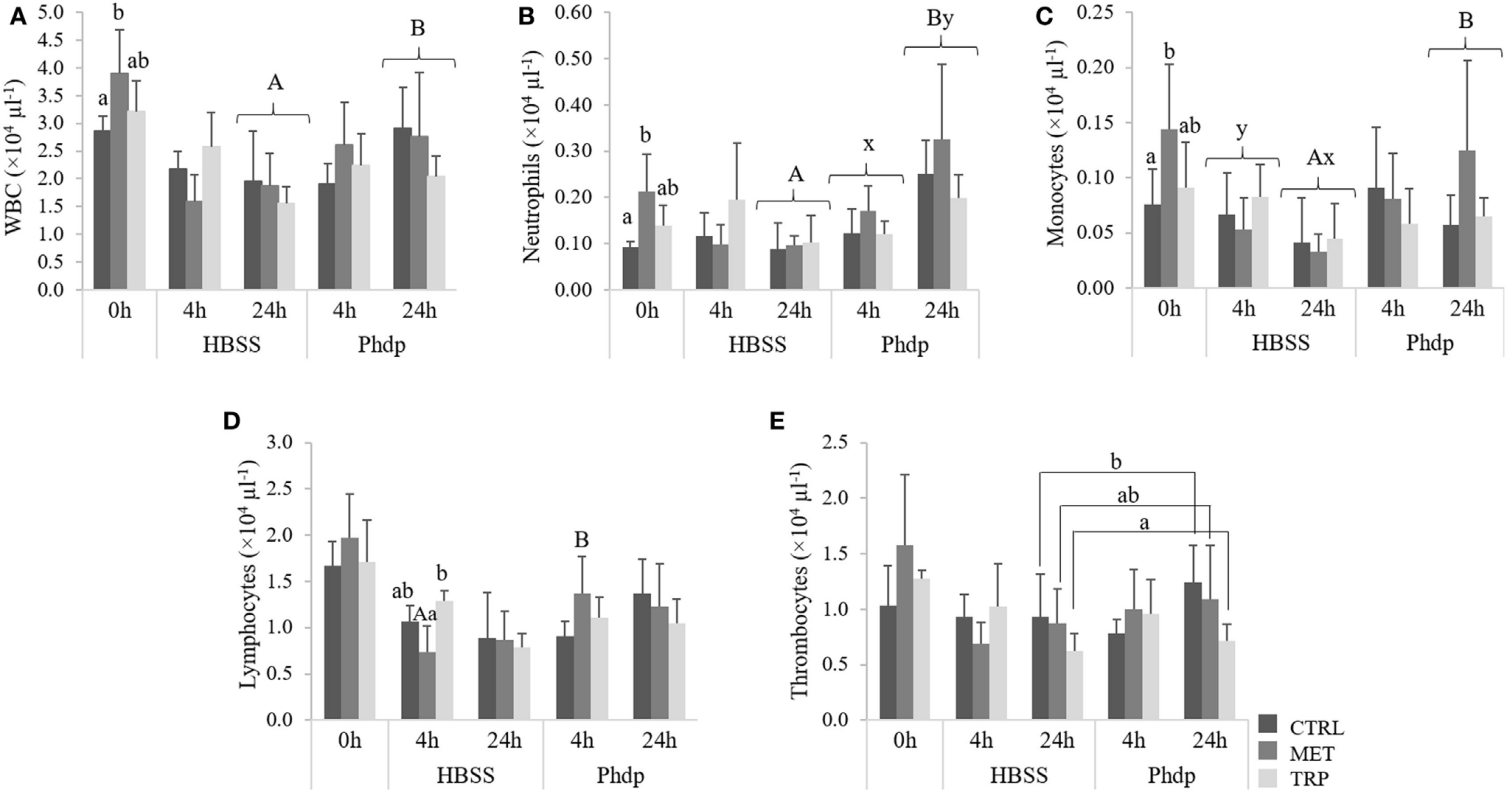
Peripheral white blood cells [white blood cells (WBC), **(A)**], neutrophils **(B)**, monocytes **(C)**, lymphocytes **(D)**, and thrombocytes **(E)** in juvenile European seabass fed CTRL (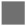), MET (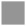), or TRP (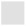) for 14 days (0 h) and sampled at 4 or 24 h post Hank’s balanced salt solution (HBSS) or *Phdp* i.p. injection (mean ± SD, *n* = 6). x and y stand for significant differences attributed to sampling time. a and b stand for significant differences between dietary treatments. Panels **(A,B)** stand for significant differences between stimuli. One-way analysis of variance (ANOVA) (before i.p. injection) and multifactorial ANOVA (after i.p. injection); Tukey *post hoc* test; *p* ≤ 0.05.

At the end of the feeding trial, total WBC, neutrophils, and monocytes concentrations were higher in fish fed MET compared to CTRL-fed animals (Figures 7A–C, respectively). At 24 h post-injection, total WBC, neutrophils, and monocytes were higher in *Phdp*-injected fish than in fish injected with HBSS (Figures 7A–C, respectively). Neutrophils increased in time in *Phdp*-injected fish, regardless of dietary treatment (Figure 7B), and monocytes concentration was lower at 24 h in HBSS-injected fish (Figure 7C). MET-fed fish showed higher lymphocyte numbers when injected with *Phdp*, compared to those injected with HBSS (Figure 7D). Furthermore, at 4 h post HBSS injection, lymphocytes were higher in TRP-fed compared to MET-fed fish (Figure 7C). At 24 h post-injection, and regardless of stimuli, thrombocytes were lower in TRP-fed fish than in CTRL-fed fish (Figure 7E).

##### Peritoneal Cavity

**Figure 8:**
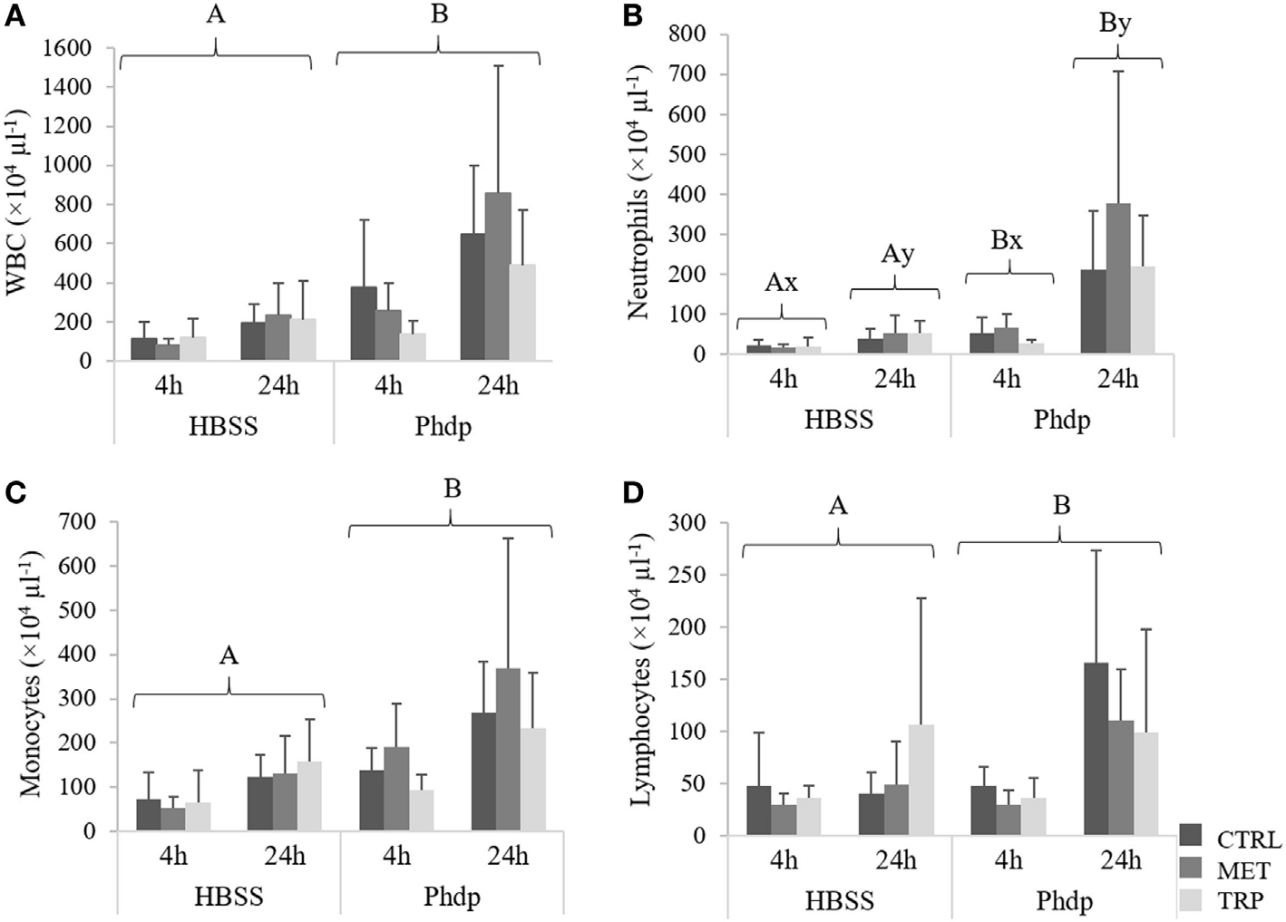
Peritoneal white blood cells [white blood cells (WBC), **(A)**], neutrophils **(B)**, monocytes **(C)**, and lymphocytes **(D)** in juvenile European seabass fed CTRL (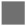), MET (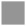), or TRP (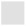) for 14 days and sampled at 4 or 24 h post Hank’s balanced salt solution (HBSS) or *Phdp* i.p. injection (mean ± SD, *n* = 6). x and y stand for significant differences between sampling times. Panels **(A,B)** stand for significant differences between stimuli. Multifactorial analysis of variance; Tukey *post hoc* test; *p* ≤ 0.05.

Total WBC, monocytes, and lymphocytes concentrations were higher in *Phdp*-injected fish compared to the HBSS-injected group, regardless of sampling time and dietary treatment (Figures 8A,C,D, respectively). Neutrophils increased with time in both HBSS- and *Phdp*-injected groups but were lower in fish challenged with vehicle than in those challenged with *Phdp*, within each sampling time (Figure 8B).

#### Quantitative Gene Expression – HK

**Figure 9:**
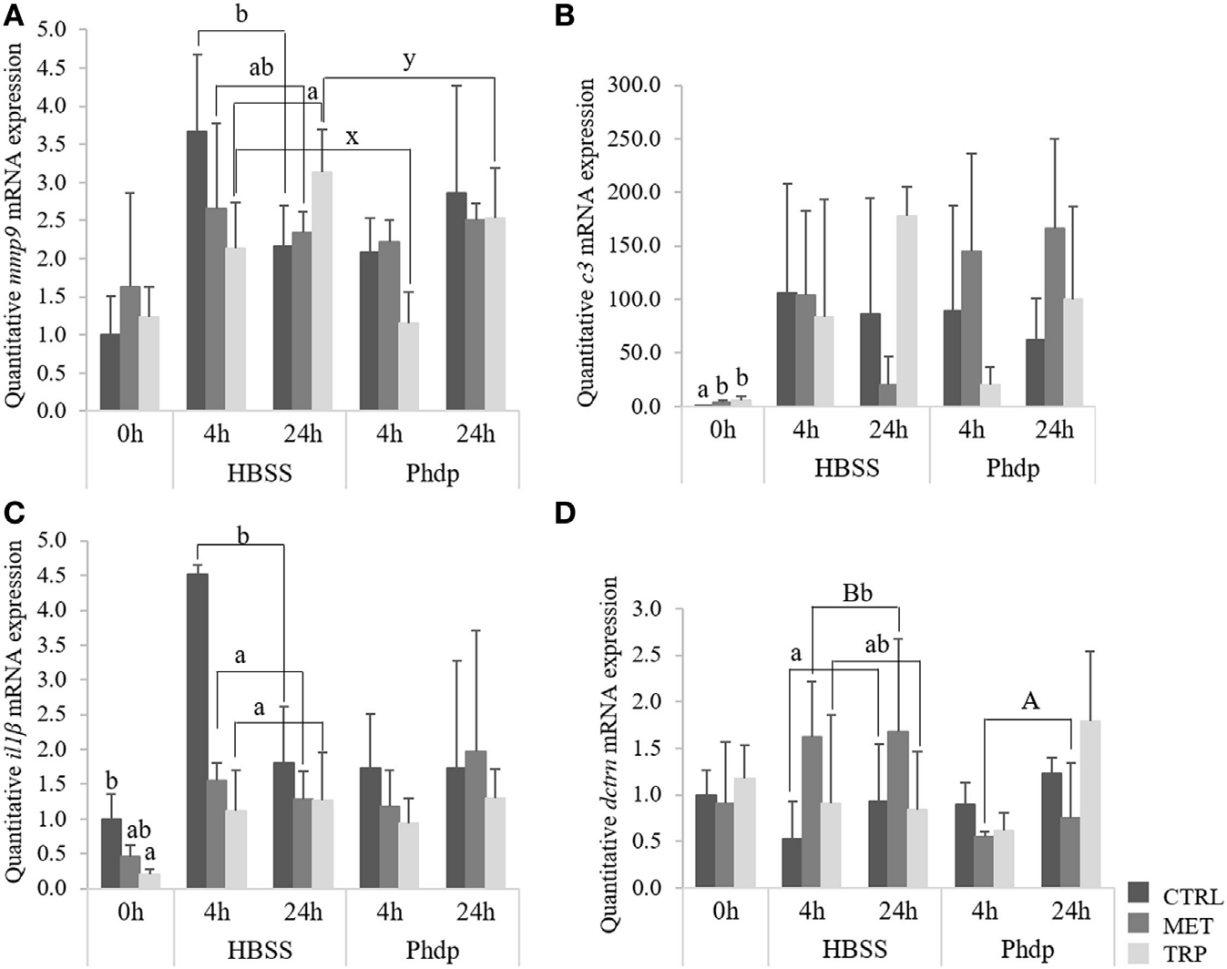
Quantitative expression of immune-related genes in the head-kidney **(A,B)** and blood **(C,D)** in juvenile European seabass fed CTRL (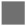), MET (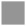), or TRP (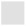) for 14 days (0 h) and sampled at 4 or 24 h post Hank’s balanced salt solution (HBSS) or *Phdp* i.p. injection (mean ± SD, *n* = 6). Metalloproteinase 9 [*mmp9*, **(A)**], complement factor 3 [*c3*, **(B)**] interleukin-1β [*il1*β, **(C)**], and dicentracin [*dctrn*, **(D)**]. x and y denote significant differences attributed to sampling time. a and b stand for significant differences between dietary treatments. **(A,B)** stand for significant differences between stimuli. One-way ANOVA (before i.p. injection) and Multifactorial ANOVA (after i.p. injection); Tukey *post hoc* test; *p* ≤ 0.05.

Gene expression of *mmp9* increased with time in TRP-fed fish, irrespectively of stimulation (Figure 9A). However, TRP-fed group presented lower mRNA levels of *mmp9* compared to CTRL-fed fish, but only when injected with HBSS (Figure 9A). *c3* expression levels increased at the end of the feeding trial in fish fed TRP and MET compared to the CTRL group and, although not statistically significant, tended to be upregulated in MET-fed fish injected with *Phdp* (Figure 9B). Gene expression of both *hep* and *il1*β was not modulated by any condition tested (Table S5 in Supplementary Material).

#### Quantitative Gene Expression—Blood

At the end of the feeding trial, *il1*β gene expression was downregulated in fish fed TRP relatively to CTRL-fed fish (Figure 9C) but no further effects were observed in any other gene expression evaluated (Table S5 in Supplementary Material). *il1*β gene expression was lower in both MET-fed and TRP-fed fish at 4 h post-injection regardless of stimulation, compared to CTRL-fed fish (Figure 9C). Upon HBSS injection, *dctrn* was upregulated in MET-fed fish relatively to their CTRL counterparts and to the same dietary group injected with *Phdp* (Figure 9D).

## Discussion

The neuroendocrine–immune network is essential for restoring homeostasis upon distress and infection. Sustaining a balanced immune response that effectively clear pathogens while minimizing damage to the host is essential. It is now recognized that in teleosts the neuroendocrine and immune systems interact in a bi-directional way, similarly to that known for mammals (4). However, this bi-directional communication has implications in physiological homeostasis and health that are yet far from being elucidated. Therefore, the present study aimed to contribute to this endeavor using a combination of a well-established inflammatory model plus a dietary intervention to assess the interaction of brain neurotransmitters, plasma cortisol levels, leukocyte migration dynamics and transcript levels of endocrine and immune-related genes. The modulation of those intimate interactions between endocrine and immune systems by amino acids would be of major assistance to promote host health.

Outcomes from the present study indicate that i.p.-injected fish (either with HBSS or UV-killed *Phdp*) were mounting a neuroendocrine response, as plasma cortisol levels increased following i.p. injection in both groups. Also, Figure S1 in Supplementary Material clearly shows in all dietary treatments an increase of serotonergic activity in i.p. injected fish compared to non-injected fish. This is in agreement with previous studies, both in mammals and teleosts, which showed a cortisol-induced production after injection challenge [i.e., bacterial lipopolysaccharide (LPS) or bacteria] (33–35). For instance, LPS induced cortisol secretion in gilthead seabream (*Sparus aurata*) at 2 and 6 h after LPS injection (36). In the present study, cortisol increase was much higher in fish fed MET and TRP than in CTRL-fed fish, and this was especially evident 24 h post-injection. Post-injection period was also characterized by lower *gr* gene expression particularly in blood of MET groups, but also in TRP groups. Similarly, the number of glucocorticoid receptors in carp decreased with increasing cortisol levels (37). The authors suggested that this response could be related to mechanisms of negative feedback in which immune cells may become resistant to cortisol. Hypothalamus–pituitary–interrenal (HPI) axis activation induces pituitary secretion of adrenocorticotropic hormone that, in turn, induces cortisol production in HK interrenal cells through activation of *mc2r* cell receptor (38). In our study, though amino acid supplementation did not seemed to affect *mc2r* expression levels, together with HK *gr*, it was downregulated at 24 h post-injection, in accordance with an established negative-feedback mechanism.

Blood *gr* downregulation in supplemented diets is absent before the challenge, being more evident 4 h after the fish were i.p. injected. In contrast, levels of cortisol in fish fed supplemented diets were much higher (2×, see Figure 4) at 4 h post-injection than before the immune challenge, whereas such increase was not observed in the CTRL group. This seems to indicate that although cortisol increase is a consequence of the HPI axis activation, apparently triggered by the i.p. injection itself as denoted by HBSS-injected fish, its release to the blood is enhanced by the presence of amino acid supplementation. This increase in cortisol at 4 h in groups fed amino acid-supplemented diets, comparatively to basal levels, might be related to two triggering mechanisms: (i) an enhanced inflammatory response driven by amino acid surplus or (ii) increased 5-HT precursor (tryptophan) which would promote synthesis of this monoamine that, in turn, would potentiate a neuroendocrine response. According to Verburg-van Kemenade et al. (4), an inflammatory response activates the central nervous system by means of circulating pathogen-associated molecular patterns, cytokines, and prostaglandins. These mediators then activate neuronal receptors that are directly involved in HPI axis activation (39). Moreover, as highlighted by Tort (40), other aspects strongly denote such intimate connection: several immune and endocrine mediators share a common phylogeny and as the HK has both endocrine and lymphoid tissues and functions, this allows a close and direct communication between both systems. Considering the significantly higher amount of total circulating leukocytes in both peripheral blood and peritoneal cavity in animals fed MET compared to other groups, it seems that i.p. injection together with enhanced leukocytes proliferation increased cortisol production in MET-fed fish. Such increase might be the result of regulatory mechanisms triggered by a significant increase on circulating immune cells. This provides further evidence on the bi-directional communication between neuroendocrine and immune systems in teleosts. In TRP-fed animals, higher cortisol production might have resulted from the increased 5-HT production, as observed in TRP-fed fish. Nonetheless, results are quite intriguing, especially for tryptophan, which has been shown to reduce cortisol response to different stressors (19, 41). The fact that cortisol levels increased with TRP instead of decreasing might be associated to the nature of the stressor. In this study, the stressor used was not a physical stressor, and foreign substances in the peritoneal cavity may directly activate immune processes that affect the HPI axis.

The involvement of amino acids in the immune response has been studied mostly in mammalian models but the interest on developing functional feeds for farmed fish raised attention on the potential of amino acids as additives for fish feed (12, 42–45). Methionine is precursor of *S*-adenosylmethionine and cysteine which narrows the topics of studies on methionine potential as feed ingredient to polyamine turnover, methylation pathways and oxidative stress (12, 46–48). Nonetheless, modulation of polyamine turnover and glutathione biosynthesis also affect the innate immune response, as it is mostly mediated by cells and characterized by intensive production of oxygen free radicals (49). Thus, as precursor of glutathione, a free radical scavenger, methionine could modulate oxidative status (50). Moreover, being necessary to polyamine biosynthesis, it may improve cell proliferation and differentiation (51). Results of the present study show an improved cellular proliferation and migration to the inflammatory focus in MET-fed fish, which is reflected in the peripheral and peritoneal leukocyte numbers. However, effects of this dietary methionine supplementation on immune response are rather ambiguous. Methionine supplementation induced HK *gpx* gene expression before the immune challenge. Being precursor of cysteine, methionine might have potentiated biosynthesis of glutathione, a co-factor in *gpx* activity. Increased enzyme production would then facilitate antioxidant activity during the inflammatory response. *gpx* gene expression was downregulated in the blood of MET-fed fish which might result from a negative-feedback mechanism in response to the earlier strong enzyme synthesis that seemed to take place before challenge. Contrary to what was expected, data on TAC during the inflammatory response does not depict an increase of the antioxidant power in MET-fed fish. Still, it is important to bear in mind that glutathione is only one among many other antioxidant molecules whose activity is also quantified by the technique used. Therefore, glutathione increase was perhaps not high enough to be detected within all other antioxidants’ activities.

Despite the fact that the cell migration indicator *mmp9* was not significantly affected by methionine supplementation, and the pro-inflammatory interleukin *il1*β was *indeed* downregulated, peripheral blood cells increased in fish fed MET. As precursor of polyamines, methionine might have promoted polyamine biosynthesis and thereby increase immune cell numbers.

The role tryptophan played in brain monoamine content was far more evident than that of methionine. The majority of neurotransmitters analyzed and their metabolites were more abundant in the optic tectum and telencephalon of animals fed TRP which probably contributed to boost cortisol production. The presence of serotonergic (52) and glucocorticoid receptors (53) in immune-related cells, as well as cytokine receptors in brain cells (54) clearly demonstrate the presence of signaling pathways established between the two systems. In addition, the fact that interrenal cells are located in an important lymphoid organ as the HK also evidences the closeness between the two systems. Chronic stress exposure has long been associated to immunosuppression and poorer disease resistance (55, 56) as reviewed by Tort (40). The present experiment was not designed to inflict chronic stress but fish might have perceived the injection itself as a neuroendocrine stimulus that triggered cortisol production. These conditions might have partly influenced the immune response.

Before being stimulated, TRP-fed fish had larger amounts of *gpx* and complement factor *c3* mRNA compared to fish fed CTRL. On the other hand, *il1*β gene expression was downregulated and peripheral leukocytes respiratory burst tended to be lower than that in CTRL-fed fish. After immune stimulation tryptophan supplementation inhibited blood *il1*β and HK *mmp9* expression and decreased thrombocytes compared to fish fed a basal diet. Tryptophan immune-suppressive effects are likely to result not only of the higher cortisol levels but also of the metabolic pathway mediated by IDO, which in mammals is known to be induced by immune stimulation, converting tryptophan to molecules with anti-inflammatory properties (8, 16, 57). Quinolinate, for instance, is able to inhibit T-cell proliferation and reduce apoptosis cytotoxicity, while immature lymphocytes develop into regulatory T-cells (16). This could help to explain the downregulation of pro-inflammatory genes and the lower numbers of circulating and peritoneal leukocytes observed in fish fed TRP. Tryptophan failed to extend the inflammatory response of seabass on plasma humoral parameters, as it suppressed the activity of peroxidase and nitric oxide synthesis (51). Cytotoxicity of the tryptophan metabolite 3-hydroxykynurenine is known to be mediated by hydrogen peroxide (58, 59). Hence, the tryptophan load and consequent metabolite production might have led to increased hydrogen peroxide production that, in turn, induced glutathione peroxidase synthesis, as observed in TRP-fed fish before i.p.-injection.

In conclusion, feeding seabass methionine- or tryptophan-supplemented diets for 2 weeks seems to modulate the neuroendocrine response during an inflammatory insult as it fostered plasma cortisol levels once fish were subjected to peritoneal inflammation. Changes in brain monoamine content were poorly affected by MET but were evident in TRP-fed fish, which also seem to be related to the observed stress response. Results point out at enhancing effects on innate immune response in MET-fed fish, as evidenced by an enhanced leukocyte response. Differently, tryptophan supplementation did not significantly change inflammatory markers, though immune-related gene expression was often downregulated. Future studies on polyamine and cytokine quantification should be considered to further characterize each amino acid role during the inflammatory response in fish.

## Ethics Statement

The experiments were approved by the Animal Welfare Committee of the Interdisciplinary Centre of Marine and Environmental Research and carried out in a registered installation (N16091.UDER). Experiments were performed by trained scientists in full compliance with national rules and following the European Directive 2010/63/EU of the European Parliament and the European Union Council on the protection of animals used for scientific purposes.

## Author Contributions

RA, MM, AA, LT, AO-T, and BC conceived the experiments, MM and RA conducted the main experimental work, and RA wrote the manuscript under the supervision of AO-T, LT, and BC, who also contributed with both reagents and goods. CF-C and FR-L gave support and counseling on molecular analysis. MC-S, MG, JM, and JS analyzed brain monoamine content. EK and SW performed liver analysis, and HP determined the amino acid composition of the experimental diets. All authors contributed to and approved the manuscript.

## Conflict of Interest Statement

The authors declare that the research was conducted in the absence of any commercial or financial relationships that could be construed as a potential conflict of interest.
