# Supplementary material for: Neuroendocrine and Immune Responses Undertake Different Fates following Tryptophan or Methionine Dietary Treatment: Tales from a Teleost Model
Source: Front Immunol. 2017 Sep 27;8:1226. doi: 10.3389/fimmu.2017.01226 (PMC5623689; doi:10.3389/fimmu.2017.01226)
Supplement: Supplementary file 1 [file data_sheet_1.docx]

***Supplementary Material***

**Neuroendocrine and immune responses to inflammation engaged different fates following tryptophan or methionine treatment: tales from a teleost model**

R. Azeredo^1,2*^, M. Machado^1,3^, A. Afonso^1,3^, C. Fierro-Castro^4^, F. Reyes-Lopez^4^, L. Tort^4^, M. Gesto^5§^, M. Conde-Sieira^1,5^, J.M. Míguez^5^, J.L. Soengas^5^, E. Kreuz^6^, S. Wuertz^6^ H. Peres^1^, A. Oliva-Teles^1,2^ and B. Costas^1,3*^

***Corresponding authors:**

Rita Azeredo and Benjamín Costas

Centro Interdisciplinar de Investigação Marinha e Ambiental (CIIMAR), Novo Edifício do Terminal de Cruzeiros do Porto de Leixões, 4450-208 Matosinhos, Portugal

Tel. +351 223401850; fax +351 223401838; Email addresses: mleme@ciimar.up.pt; bcostas@ciimar.up.pt

Supplementary table 1. Hypothalamic monoamine content in juvenile European seabass fed dietary treatments for 14 days (0 h) and sampled at 4 h or 24 h post HBSS- or *Phdp-* i.p. injection.

| Hypothalamic monoamine content | | | | | | | | | | | | | | | | | | | | | | | | |
| --- | --- | --- | --- | --- | --- | --- | --- | --- | --- | --- | --- | --- | --- | --- | --- | --- | --- | --- | --- | --- | --- | --- | --- | --- |
|  |  |  | NA | | | DA | | | DOPAC | | | 5-HT | | | 5HIAA | | | DOPAC/DA | | | 5HIAA/5HT | | | |
|  | 0 h | CTRL | 44.5 ab | ± | 7.0 | 8.2 | ± | 0.5 | 0.7 | ± | 0.4 | 39.9 | ± | 6.9 | 5.2 | ± | 1.7 | 8.5 | ± | 4.2 | 12.7 | ± | 2.8 |  |
|  |  | MET | 56.1 b | ± | 12.3 | 10.6 | ± | 1.9 | 0.9 | ± | 0.4 | 49.2 | ± | 9.3 | 6.5 | ± | 2.2 | 8.2 | ± | 2.3 | 12.0 | ± | 1.8 |  |
|  |  | TRP | 40.1 a | ± | 7.4 | 8.8 | ± | 1.4 | 0.8 | ± | 0.3 | 37.0 | ± | 10.6 | 5.9 | ± | 2.6 | 9.2 | ± | 1.8 | 15.8 | ± | 3.8 |  |
| CTRL | HBSS | 4 h | 42.4 | ± | 10.9 | 9.6 | ± | 1.6 | 0.9 | ± | 0.3 | 42.0 | ± | 11.6 | 7.9 | ± | 1.9 | 9.4 | ± | 1.3 | 19.4 | ± | 3.6 |  |
|  |  | 24 h | 40.5 | ± | 7.3 | 8.6 | ± | 3.2 | 0.9 | ± | 0.4 | 44.3 | ± | 15.2 | 8.2 | ± | 3.7 | 12.6 | ± | 1.9 | 16.9 | ± | 2.9 |  |
|  | *Phdp* | 4 h | 38.2 | ± | 8.9 | 7.2 | ± | 2.4 | 0.7 | ± | 0.3 | 36.3 | ± | 11.2 | 6.3 | ± | 3.1 | 10.0 | ± | 3.8 | 17.7 | ± | 3.5 |  |
|  |  | 24 h | 43.2 | ± | 6.8 | 8.4 | ± | 2.6 | 0.8 | ± | 0.2 | 41.6 | ± | 7.8 | 7.9 | ± | 1.9 | 9.2 | ± | 0.2 | 19.4 | ± | 1.8 |  |
| MET | HBSS | 4 h | 44.9 | ± | 8.9 | 8.0 | ± | 3.4 | 0.9 | ± | 0.5 | 40.8 | ± | 13.0 | 7.5 | ± | 2.8 | 13.1 | ± | 2.5 | 18.4 | ± | 2.4 |  |
|  |  | 24 h | 45.1 | ± | 13.4 | 9.1 | ± | 1.7 | 0.8 | ± | 0.4 | 43.2 | ± | 10.3 | 6.6 | ± | 1.5 | 8.7 | ± | 2.5 | 15.0 | ± | 0.9 |  |
|  | *Phdp* | 4 h | 46.7 | ± | 8.1 | 10.2 | ± | 1.6 | 0.9 | ± | 0.2 | 43.4 | ± | 6.3 | 7.3 | ± | 1.6 | 9.7 | ± | 0.9 | 18.0 | ± | 2.6 |  |
|  |  | 24 h | 46.7 | ± | 6.8 | 9.8 | ± | 1.9 | 1.0 | ± | 0.2 | 47.4 | ± | 8.9 | 8.3 | ± | 3.1 | 10.8 | ± | 1.0 | 15.9 | ± | 2.2 |  |
| TRP | HBSS | 4 h | 51.1 | ± | 8.3 | 9.8 | ± | 1.5 | 1.2 | ± | 0.3 | 48.2 | ± | 4.7 | 9.1 | ± | 0.9 | 12.4 | ± | 1.3 | 19.1 | ± | 2.4 |  |
|  |  | 24 h | 41.6 | ± | 4.0 | 9.2 | ± | 1.1 | 0.9 | ± | 0.2 | 45.6 | ± | 6.2 | 8.0 | ± | 1.4 | 9.9 | ± | 0.6 | 16.6 | ± | 2.6 |  |
|  | *Phdp* | 4 h | 48.2 | ± | 9.6 | 9.2 | ± | 3.1 | 1.1 | ± | 0.6 | 43.5 | ± | 14.0 | 9.0 | ± | 3.9 | 10.3 | ± | 2.8 | 22.2 | ± | 4.1 |  |
|  |  | 24 h | 43.7 | ± | 5.4 | 8.8 | ± | 3.8 | 1.1 | ± | 0.4 | 45.9 | ± | 11.1 | 8.0 | ± | 1.3 | 11.3 | ± | 3.2 | 16.6 | ± | 1.5 |  |

| **Multifactorial ANOVA** | **P-value** | | | | | | | Time | |  |
| --- | --- | --- | --- | --- | --- | --- | --- | --- | --- | --- |
|  | time | diet | stimulus | time × diet | time × stimuli | diet × stimuli | diet × stimuli × time | 4 h | 24 h | |
| NA | NS | NS | NS | NS | NS | NS | NS |  |  | |
| DA | NS | NS | NS | NS | NS | NS | NS |  |  | |
| DOPAC | NS | NS | NS | NS | NS | NS | NS |  |  | |
| 5-HT | NS | NS | NS | NS | NS | NS | NS |  |  | |
| 5HIAA | NS | NS | NS | NS | NS | NS | NS |  |  | |
| DOPAC/DA ratio | NS | NS | NS | NS | NS | NS | NS |  |  | |
| 5HIAA/5HT ratio | 0.001 | NS | NS | NS | NS | NS | NS | y | x | |

| **One-way ANOVA** | **P-value** |
| --- | --- |
|  | diet |
| NA | 0.025 |
| DA | NS |
| DOPAC | NS |
| 5-HT | NS |
| 5HIAA | NS |
| DOPAC/DA ratio | NS |
| 5HIAA/5HT ratio | NS |

Values are expressed as means ± standard error (n=6). Noradrenaline (NA), dopamine (DA), 3,4-dihydroxyphenylacetic acid (DOPAC), serotonin (5-HT) and 5-hydroxyindoleacetic acid (5-HIAA). x and y identify significant differences between sampling time. a and b stand for significant differences between dietary treatments. One-way ANOVA (before i.p. injection) and Multifactorial ANOVA (after i.p. injection); Tukey post hoc test; p≤0.05).

Supplementary table 2. Telencephalic monoamine content in juvenile European seabass fed dietary treatments for 14 days (0 h) and sampled at 4 h or 24 h post HBSS- or *Phdp-* i.p. injection.

| Telencephalic monoamine content | | | | | | | | | | | | | | | | | | | | | | | | | | | | | |  |
| --- | --- | --- | --- | --- | --- | --- | --- | --- | --- | --- | --- | --- | --- | --- | --- | --- | --- | --- | --- | --- | --- | --- | --- | --- | --- | --- | --- | --- | --- | --- |
|  |  |  | NA | | | | DA | | | DOPAC | | | | 5-HT | | | | 5HIAA | | | | DOPAC/DA | | | | 5HIAA/5HT | | | | |
|  | 0 h | CTRL | 27.1 | ± | 1.5 | 3.3 | | ± | 0.2 | | 0.5 | ± | 0.3 | 42.5 | ± | 6.1 | 13.0 | | ± | 1.5 | 14.9 | | ± | 9.4 | 30.9 | | ± | 4.5 |  |  |
|  |  | MET | 25.2 | ± | 10.0 | 2.3 | | ± | 1.6 | | 0.4 | ± | 0.3 | 31.8 | ± | 17.7 | 10.6 | | ± | 5.7 | 16.3 | | ± | 10.6 | 33.4 | | ± | 5.3 |  |  |
|  |  | TRP | 26.6 | ± | 3.1 | 3.5 | | ± | 1.2 | | 0.5 | ± | 0.3 | 50.0 | ± | 14.9 | 17.0 | | ± | 4.0 | 14.2 | | ± | 4.7 | 34.9 | | ± | 5.3 |  |  |
| CTRL | HBSS | 4 h | 29.5 | ± | 3.7 | 4.1 y | | ± | 0.9 | | 0.6 | ± | 0.2 | 49.3 | ± | 9.8 | 18.5 | | ± | 3.8 | 15.8 | | ± | 3.7 | 37.8 | | ± | 5.0 |  |  |
|  |  | 24 h | 26.9 | ± | 4.4 | 2.6 x | | ± | 0.3 | | 0.5 | ± | 0.2 | 44.6 | ± | 8.2 | 16.3 | | ± | 3.2 | 18.0 | | ± | 1.5 | 36.5 | | ± | 1.9 |  |  |
|  | *Phdp* | 4 h | 27.0 | ± | 3.7 | 3.1 | | ± | 0.3 | | 0.4 | ± | 0.1 | 38.4 | ± | 9.3 | 14.3 | | ± | 4.3 | 14.2 | | ± | 3.6 | 37.0 | | ± | 6.0 |  |  |
|  |  | 24 h | 27.8 | ± | 3.8 | 3.8 | | ± | 1.1 | | 0.4 | ± | 0.1 | 47.5 | ± | 7.7 | 16.9 | | ± | 3.0 | 11.6 | | ± | 2.3 | 35.5 | | ± | 1.5 |  |  |
| MET | HBSS | 4 h | 28.3 | ± | 4.5 | 4.1 | | ± | 0.8 | | 0.6 | ± | 0.2 | 45.2 | ± | 14.3 | 16.4 | | ± | 4.9 | 13.4 | | ± | 4.1 | 36.6 | | ± | 5.2 |  |  |
|  |  | 24 h | 27.3 | ± | 5.7 | 3.2 | | ± | 0.9 | | 0.5 | ± | 0.1 | 41.3 | ± | 8.3 | 15.5 | | ± | 3.6 | 13.2 | | ± | 2.7 | 37.4 | | ± | 3.4 |  |  |
|  | *Phdp* | 4 h | 26.5 | ± | 6.1 | 3.3 | | ± | 0.7 | | 0.4 | ± | 0.1 | 42.8 | ± | 12.0 | 15.0 | | ± | 4.6 | 14.7 | | ± | 5.9 | 34.9 | | ± | 1.9 |  |  |
|  |  | 24 h | 28.2 | ± | 4.6 | 2.8 | | ± | 0.8 | | 0.3 | ± | 0.0 | 41.5 | ± | 5.1 | 14.7 | | ± | 2.9 | 13.4 | | ± | 3.8 | 35.3 | | ± | 3.9 |  |  |
| TRP | HBSS | 4 h | 26.1 | ± | 2.7 | 3.8 | | ± | 0.4 | | 0.5 | ± | 0.2 | 53.9 | ± | 9.8 | 19.0 | | ± | 4.2 | 13.7 | | ± | 3.0 | 35.1 | | ± | 4.0 |  |  |
|  |  | 24 h | 26.1 | ± | 5.4 | 3.1 | | ± | 0.3 | | 0.5 | ± | 0.1 | 50.0 | ± | 10.6 | 17.3 | | ± | 1.0 | 16.5 | | ± | 2.8 | 34.3 | | ± | 4.2 |  |  |
|  | *Phdp* | 4 h | 26.5 | ± | 4.1 | 3.3 | | ± | 0.4 | | 0.6 | ± | 0.3 | 48.5 | ± | 8.2 | 18.8 | | ± | 4.6 | 19.7 | | ± | 9.0 | 38.3 | | ± | 4.4 |  |  |
|  |  | 24 h | 27.1 | ± | 4.6 | 2.8 | | ± | 0.4 | | 0.4 | ± | 0.0 | 48.1 | ± | 10.1 | 18.9 | | ± | 0.8 | 17.6 | | ± | 3.3 | 37.1 | | ± | 6.1 |  |  |

|  | | | | | | | | | | | | | | | | | |  | diet × stimuli | | | | | |  |
| --- | --- | --- | --- | --- | --- | --- | --- | --- | --- | --- | --- | --- | --- | --- | --- | --- | --- | --- | --- | --- | --- | --- | --- | --- | --- |
| **Multifactorial ANOVA** | **P-value** | | | | | | |  | time | |  | diet | | |  | stimuli | |  | HBSS | | | *Phdp* | | |  |
|  | time | diet | stimulus | time × diet | time × stimuli | diet × stimuli | diet × stimuli × time |  | 4 h | 24 h |  | CTRL | MET | TRP |  | HBSS | *Phdp* |  | CTRL | MET | TRP | CTRL | MET | TRP | |
| NA | NS | NS | NS | NS | NS | NS | NS |  |  |  |  |  |  |  |  |  |  |  |  |  |  |  |  |  | |
| DA | 0.001 | NS | NS | NS | 0.005 | NS | 0.023 |  |  |  |  |  |  |  |  |  |  |  |  |  |  |  |  |  | |
| DOPAC | 0.04 | NS | 0.01 | NS | NS | NS | NS |  | y | x |  |  |  |  |  | B | A |  |  |  |  |  |  |  | |
| 5-HT | NS | 0.03 | NS | NS | NS | NS | NS |  |  |  |  | ab | a | b |  |  |  |  |  |  |  |  |  |  | |
| 5HIAA | NS | 0.03 | NS | NS | NS | NS | NS |  |  |  |  | ab | a | b |  |  |  |  |  |  |  |  |  |  | |
| DOPAC/DA ratio | NS | 0.04 | NS | NS | NS | 0.024 | NS |  |  |  |  |  |  |  |  |  |  |  |  |  |  | a | ab | b | |
| 5HIAA/5HT ratio | NS | NS | NS | NS | NS | NS | NS |  |  |  |  |  |  |  |  |  |  |  |  |  |  |  |  |  | |

| **One-way ANOVA** | **P-value** |
| --- | --- |
|  | Diet |
| NA | NS |
| DA | NS |
| DOPAC | NS |
| 5-HT | NS |
| 5HIAA | NS |
| DOPAC/DA ratio | NS |
| 5HIAA/5HT ratio | NS |

Values are expressed as means ± standard error (n=6). Noradrenaline (NA), dopamine (DA), 3,4-dihydroxyphenylacetic acid (DOPAC), serotonin (5-HT) and 5-hydroxyindoleacetic acid (5-HIAA). x and y identify significant differences between sampling times. a and b stand for significant differences attributed to dietary treatments. A and B identify significant differences between stimuli. One-way ANOVA (before i.p. injection) and Multifactorial ANOVA (after i.p. injection); Tukey post hoc test; p≤0.05).

Supplementary table 3. Optic tectum monoamine content in juvenile European seabass fed dietary treatments for 14 days (0 h) and sampled at 4 h or 24 h post HBSS- or *Phdp-* i.p. injection.

| Optic tectum monoamine content | | | | | | | | | | | | | | | | | | | | | | | | | | | |
| --- | --- | --- | --- | --- | --- | --- | --- | --- | --- | --- | --- | --- | --- | --- | --- | --- | --- | --- | --- | --- | --- | --- | --- | --- | --- | --- | --- |
|  |  |  | NA | | | DA | | | | DOPAC | | | 5-HT | | | 5HIAA | | | | DOPAC/DA | | | | 5HIAA/5HT | | |  |
|  | 0 h | CTRL | 22.6 | ± | 8.5 | | 2.7 | ± | 0.9 | 1.1 | ± | 0.3 | 19.9 | ± | 5.4 | | 3.9 | ± | 1.1 | 42.7 | ± | 9.3 | 19.2 | | ± | 1.4 |  |
|  |  | MET | 22.8 | ± | 3.5 | | 2.9 | ± | 0.7 | 1.3 | ± | 0.5 | 24.5 | ± | 7.2 | | 5.0 | ± | 1.6 | 43.7 | ± | 12.7 | 20.5 | | ± | 3.3 |  |
|  |  | TRP | 20.7 | ± | 3.6 | | 2.5 | ± | 0.5 | 1.0 | ± | 0.3 | 23.4 | ± | 5.2 | | 5.2 | ± | 1.1 | 42.0 | ± | 17.6 | 23.0 | | ± | 6.0 |  |
| CTRL | HBSS | 4 h | 23.3 | ± | 3.8 | | 2.3 | ± | 0.3 | 1.3 | ± | 0.4 | 25.7 | ± | 4.8 | | 6.0 | ± | 0.7 | 51.8 | ± | 12.3 | 23.9 | | ± | 3.2 |  |
|  |  | 24 h | 21.5 | ± | 4.8 | | 2.7 | ± | 0.6 | 1.4 | ± | 0.3 | 24.0 | ± | 4.9 | | 5.7 | ± | 1.0 | 47.7 | ± | 9.6 | 24.0 | | ± | 5.0 |  |
|  | *Phdp* | 4 h | 21.7 | ± | 5.3 | | 2.2 | ± | 0.3 | 1.1 | ± | 0.1 | 18.9 | ± | 3.4 | | 5.1 | ± | 1.2 | 46.0 | ± | 6.5 | 27.0 | | ± | 4.4 |  |
|  |  | 24 h | 21.6 | ± | 6.2 | | 2.6 | ± | 0.8 | 1.0 | ± | 0.2 | 22.8 | ± | 6.3 | | 5.9 | ± | 1.2 | 42.0 | ± | 3.8 | 26.6 | | ± | 4.1 |  |
| MET | HBSS | 4 h | 20.3 | ± | 4.2 | | 2.5 | ± | 0.3 | 1.3 | ± | 0.2 | 22.3 | ± | 6.8 | | 5.3 | ± | 1.1 | 54.0 | ± | 14.1 | 24.8 | | ± | 5.5 |  |
|  |  | 24 h | 20.9 | ± | 3.6 | | 2.7 | ± | 0.4 | 1.3 | ± | 0.1 | 20.7 | ± | 2.4 | | 5.9 | ± | 1.0 | 51.8 | ± | 13.2 | 28.8 | | ± | 6.2 |  |
|  | *Phdp* | 4 h | 19.5 | ± | 4.9 | | 2.2 | ± | 0.2 | 1.2 | ± | 0.4 | 18.9 | ± | 7.9 | | 4.4 | ± | 0.7 | 54.3 | ± | 14.6 | 25.4 | | ± | 8.4 |  |
|  |  | 24 h | 20.8 | ± | 4.6 | | 2.6 | ± | 0.2 | 1.4 | ± | 0.2 | 21.2 | ± | 2.3 | | 5.8 | ± | 0.9 | 52.4 | ± | 5.1 | 27.3 | | ± | 4.4 |  |
| TRP | HBSS | 4 h | 21.1 | ± | 2.8 | | 2.3 | ± | 0.2 | 1.1 | ± | 0.3 | 22.9 | ± | 2.8 | | 5.8 | ± | 1.0 | 46.4 | ± | 10.0 | 25.2 | | ± | 4.3 |  |
|  |  | 24 h | 20.6 | ± | 1.9 | | 2.2 | ± | 0.6 | 1.3 | ± | 0.2 | 23.3 | ± | 3.5 | | 5.6 | ± | 0.8 | 51.7 | ± | 7.5 | 24.6 | | ± | 4.8 |  |
|  | *Phdp* | 4 h | 23.0 | ± | 3.1 | | 3.0 | ± | 0.4 | 1.5 | ± | 0.1 | 27.5 | ± | 5.1 | | 6.6 | ± | 0.9 | 48.2 | ± | 8.1 | 24.2 | | ± | 3.2 |  |
|  |  | 24 h | 22.2 | ± | 3.4 | | 2.9 | ± | 0.3 | 1.5 | ± | 0.2 | 26.0 | ± | 4.9 | | 5.7 | ± | 1.2 | 50.8 | ± | 6.7 | 22.0 | | ± | 1.3 |  |

|  |  |  |  |  |  |  |  |  |  | time × diet | | | | | |  | diet × stimuli | | | | | |
| --- | --- | --- | --- | --- | --- | --- | --- | --- | --- | --- | --- | --- | --- | --- | --- | --- | --- | --- | --- | --- | --- | --- |
| **Multifactorial ANOVA** | | **P-value** | | | | | | |  | 4 h | | | 24 h | | |  | HBSS | | | *Phdp* | | |
|  |  | time | diet | stimulus | time × diet | time × stimuli | diet × stimuli | diet × stimuli × time |  | CTRL | MET | TRP | CTRL | MET | TRP |  | CTRL | MET | TRP | CTRL | MET | TRP |
| NA | | NS | NS | NS | NS | NS | NS | NS |  |  |  |  |  |  |  |  |  |  |  |  |  |  |
| DA | | NS | NS | NS | NS | NS | 0.010 | NS |  |  |  |  |  |  |  |  |  |  | A |  |  | B |
| DOPAC | | NS | NS | NS | NS | NS | 0.001 | NS |  |  |  |  |  |  |  |  | B |  |  | Aa | ab | b |
| 5-HT | | NS | 0.017 | NS | NS | NS | 0.029 | NS |  |  |  |  |  |  |  |  |  |  |  | a | a | b |
| 5HIAA | | NS | NS | NS | 0.047 | NS | NS | NS |  | ab | a | b |  |  |  |  |  |  |  |  |  |  |
| DOPAC/DA ratio | | NS | NS | NS | NS | NS | NS | NS |  |  |  |  |  |  |  |  |  |  |  |  |  |  |
| 5HIAA/5HT ratio | | NS | NS | NS | NS | NS | NS | NS |  |  |  |  |  |  |  |  |  |  |  |  |  |  |

| **One-way ANOVA** | **P-value** |
| --- | --- |
|  | Diet |
| NA | NS |
| DA | NS |
| DOPAC | NS |
| 5-HT | NS |
| 5HIAA | NS |
| DOPAC/DA ratio | NS |
| 5HIAA/5HT ratio | NS |

Values are expressed as means ± standard error (n=6). Noradrenaline (NA), dopamine (DA), 3,4-dihydroxyphenylacetic acid (DOPAC), serotonin (5-HT) and 5-hydroxyindoleacetic acid (5-HIAA). x and y identify significant differences attributed to sampling time. a and b stand for significant differences between dietary treatments. A and B identify significant differences attributed to stimuli. One-way ANOVA (before i.p. injection) and Multifactorial ANOVA (after i.p. injection); Tukey post hoc test; p≤0.05).

Supplementary table 4. Liver total antioxidant capacity in juvenile European seabass fed dietary treatments for 14 days (0 h) and sampled at 4 h or 24 h post HBSS- or *Phdp-* i.p. injection.

| Total antioxidant capacity  (mM mg^-1^ protein) | | | | | | |
| --- | --- | --- | --- | --- | --- | --- |
|  | 0 h | CTRL | 5.9 | ± | 2.5 |  |
|  |  | MET | 4.1 | ± | 2.1 |  |
|  |  | TRP | 3.1 | ± | 0.9 |  |
| CTRL | HBSS | 4 h | 3.5 | ± | 0.5 |  |
|  |  | 24 h | 7.4 | ± | 5.1 |  |
|  | *Phdp* | 4 h | 3.3 | ± | 0.9 |  |
|  |  | 24 h | 11.4 | ± | 5.9 |  |
| MET | HBSS | 4 h | 3.5 | ± | 0.2 |  |
|  |  | 24 h | 5.2 | ± | 3.3 |  |
|  | *Phdp* | 4 h | 4.5 | ± | 0.6 |  |
|  |  | 24 h | 5.0 | ± | 2.5 |  |
| TRP | HBSS | 4 h | 4.0 | ± | 1.1 |  |
|  |  | 24 h | 7.1 | ± | 4.0 |  |
|  | *Phdp* | 4 h | 3.3 | ± | 0.7 |  |
|  |  | 24 h | 5.7 | ± | 1.5 |  |

| **Multifactorial ANOVA** | **P-value** | | | | | | |  | **time** | |
| --- | --- | --- | --- | --- | --- | --- | --- | --- | --- | --- |
|  | time | diet | stimulus | time × diet | time × stimuli | diet × stimuli | diet × stimuli × time |  | 4 h | 24 h |
| Total antioxidant capacity (mM mg^-1^ protein) | 0.002 | NS | NS | NS | NS | NS | NS |  | x | y |

| **One-way ANOVA** | **P-value** |
| --- | --- |
|  | Diet |
| Total antioxidant capacity (mM mg^-1^ protein) | NS |

Values are expressed as means ± standard error (n=6). x and y stand for significant differences between sampling times. One-way ANOVA (before i.p. injection) and Multifactorial ANOVA (after i.p. injection); Tukey post hoc test; p≤0.05).

Supplementary table 5. Relative neuroendocrine- and immune-related gene expression in juvenile European seabass blood and head-kidney fed dietary treatments for 14 days (0 h) and sampled at 4 h or 24 h post HBSS- or *Phdp-* i.p. injection.

|  |  |  | Blood | | |  | Head-kidney | | | | | | | | | | | | | | |
| --- | --- | --- | --- | --- | --- | --- | --- | --- | --- | --- | --- | --- | --- | --- | --- | --- | --- | --- | --- | --- | --- |
|  |  |  | *mmp9* | | |  | *gpx* | | | *mc2r* | | | *gr* | | | *hep* | | | *il1β* | | |
|  | 0 h | CTRL | 1.0 | ± | 0.9 |  | 0.5 | ± | 1.0 | 0.8 | ± | 0.3 | 0.8 | ± | 0.2 | 1.0 | ± | 0.6 | 1.0 | ± | 0.7 |
|  |  | MET | 1.1 | ± | 0.2 |  | 15.2 | ± | 10.9 | 0.9 | ± | 0.2 | 0.7 | ± | 0.3 | 1.7 | ± | 1.1 | 1.9 | ± | 1.8 |
|  |  | TRP | 0.9 | ± | 0.7 |  | 369.1 | ± | 150.6 | 0.8 | ± | 0.2 | 0.8 | ± | 0.2 | 1.4 | ± | 1.0 | 1.1 | ± | 0.8 |
| CTRL | HBSS | 4 h | 2.3 | ± | 1.9 |  | 2968.8 | ± | 2153.6 | 0.5 | ± | 0.3 | 0.3 | ± | 0.3 | 3.8 | ± | 3.9 | 2.9 | ± | 2.4 |
|  |  | 24 h | 3.5 | ± | 1.0 |  | 403.7 | ± | 346.5 | 0.2 | ± | 0.3 | 0.1 | ± | 0.2 | 1.9 | ± | 0.8 | 0.7 | ± | 0.5 |
|  | *Phdp* | 4 h | 3.4 | ± | 1.7 |  | 3069.3 | ± | 2766.1 | 1.2 | ± | 1.0 | 0.9 | ± | 0.2 | 2.8 | ± | 3.0 | 1.7 | ± | 1.2 |
|  |  | 24 h | 3.7 | ± | 1.7 |  | 2204.3 | ± | 2307.8 | 0.3 | ± | 0.3 | 0.2 | ± | 0.3 | 2.1 | ± | 1.1 | 2.5 | ± | 1.6 |
| MET | HBSS | 4 h | 3.5 | ± | 0.6 |  | 2531.3 | ± | 2093.9 | 0.6 | ± | 0.7 | 0.9 | ± | 1.0 | 2.5 | ± | 1.5 | 2.1 | ± | 2.0 |
|  |  | 24 h | 1.6 | ± | 0.1 |  | 2544.2 | ± | 1735.3 | 0.2 | ± | 0.4 | 0.3 | ± | 0.4 | 1.2 | ± | 0.7 | 0.7 | ± | 0.2 |
|  | *Phdp* | 4 h | 2.7 | ± | 1.1 |  | 666.9 | ± | 642.1 | 0.9 | ± | 0.4 | 1.1 | ± | 1.0 | 2.3 | ± | 0.2 | 1.4 | ± | 0.5 |
|  |  | 24 h | 1.7 | ± | 0.7 |  | 3477.3 | ± | 2738.3 | 0.6 | ± | 0.6 | 0.5 | ± | 0.6 | 2.6 | ± | 2.6 | 1.4 | ± | 0.8 |
| TRP | HBSS | 4 h | 1.8 | ± | 0.3 |  | 1274.6 | ± | 1480.0 | 0.8 | ± | 0.1 | 0.5 | ± | 0.1 | 2.0 | ± | 1.2 | 1.3 | ± | 0.6 |
|  |  | 24 h | 3.8 | ± | 1.3 |  | 3954.5 | ± | 2192.4 | 0.6 | ± | 0.9 | 0.5 | ± | 0.7 | 3.0 | ± | 3.1 | 2.6 | ± | 1.8 |
|  | *Phdp* | 4 h | 1.6 | ± | 0.3 |  | 3183.4 | ± | 3052.3 | 1.1 | ± | 0.6 | 0.8 | ± | 0.4 | 3.5 | ± | 3.1 | 1.6 | ± | 0.9 |
|  |  | 24 h | 2.3 | ± | 3.4 |  | 1130.5 | ± | 1086.5 | 0.4 | ± | 0.5 | 0.3 | ± | 0.3 | 3.0 | ± | 1.5 | 2.1 | ± | 1.4 |

|  | **P-value** | | | | | | |  | time | |
| --- | --- | --- | --- | --- | --- | --- | --- | --- | --- | --- |
| **Multifactorial ANOVA** | time | diet | stimulus | time × diet | time × stimuli | diet × stimuli | diet × stimuli × time |  | 4 h | 24 h |
| *mmp9* | NS | NS | NS | NS | NS | NS | NS |  |  |  |
| *mc2r* | 0.006 | NS | NS | NS | NS | NS | NS |  | y | x |
| *gr* | 0.003 | NS | NS | NS | NS | NS | NS |  | y | x |
| *hep* | NS | NS | NS | NS | NS | NS | NS |  |  |  |
| *il1β* | NS | NS | NS | NS | NS | NS | NS |  |  |  |

| **One-way ANOVA** | | **P-value** |
| --- | --- | --- |
|  |  | Diet |
| *Blood* | *mmp9* | NS |
| *Head-kidney* | *mc2r* | NS |
|  | *gr* | NS |
|  | *hep* | NS |
|  | *il1β* | NS |

Values are expressed as means ± standard error (n=6). Metalloproteinase 9 (*mmp9*), melanocortin receptor 2 (*mc2r*), glucocorticoid receptor (*gr*), hepcidin (*hep*) and interleukin 1β (*il1β*)**.** x and y stand for significant differences between sampling times. One-way ANOVA (before i.p. injection) and Multifactorial ANOVA (after i.p. injection); Tukey post hoc test; p≤0.05).

Supplementary figure 1. Brain 5HIAA/5HT ratio in European seabass hypothalamus **(A)**, telencephalon **(B)** and optic tectum **(C)** fed different dietary treatments for 14 days (■) and sampled 4 h post i.p. injection with *Phdp* (■) or HBSS (■). Data are mean ± SD (n=6).


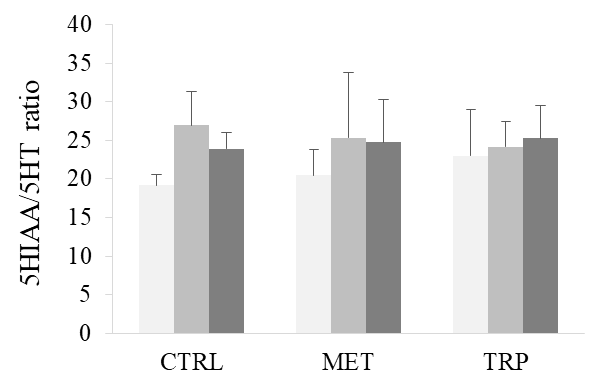

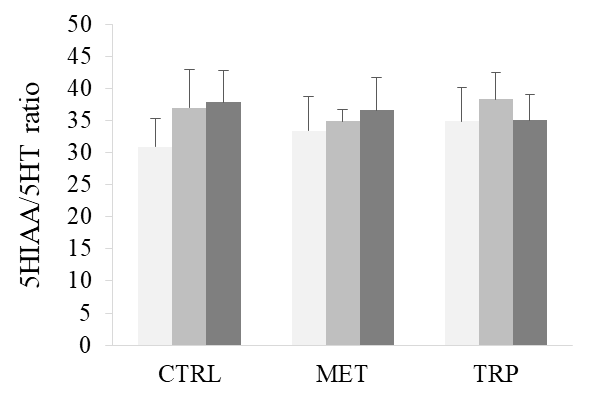

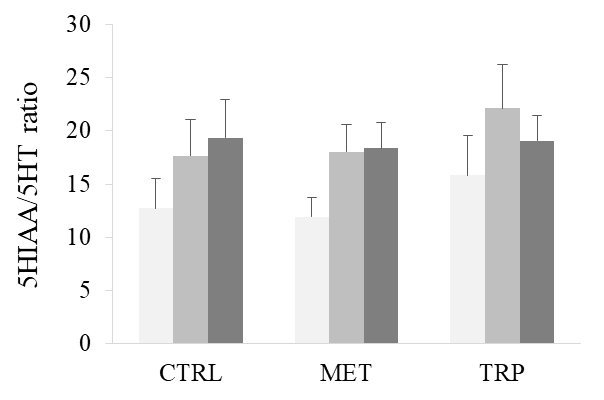


**A**

**B**

**C**
